# The Coordination Chemistry of Two Peptidic Models of NFeoB and Core CFeoB Regions of FeoB Protein: Complexes of Fe(II), Mn(II), and Zn(II)

**DOI:** 10.1021/acs.inorgchem.4c05111

**Published:** 2025-03-06

**Authors:** Bartosz Orzel, Malgorzata Ostrowska, Slawomir Potocki, Maria Antonietta Zoroddu, Henryk Kozlowski, Massimiliano Peana, Elzbieta Gumienna-Kontecka

**Affiliations:** † Faculty of Chemistry, University of Wrocław, Wrocław 50-383, Poland; ‡ Department of Chemical, Physical, Mathematical and Natural Sciences, University of Sassari, Sassari 07100, Italy; § Faculty of Health Sciences, University of Opole, Katowicka, Opole 68 45-060, Poland

## Abstract

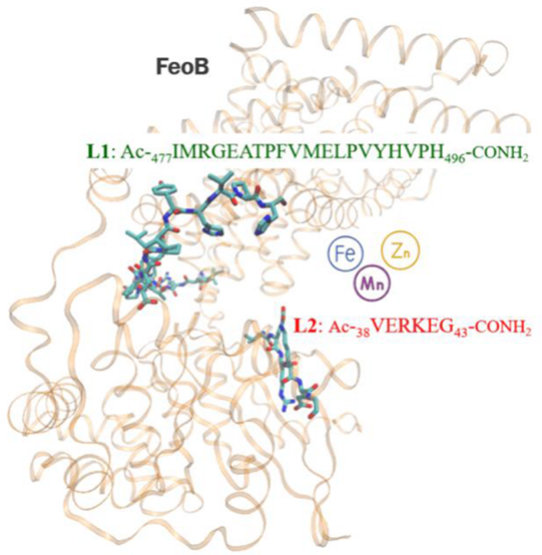

Often necessary for efficient Fe(II) trafficking into bacterial cell, the Feo system is a vital transporter for many pathogenic bacteria and indispensable for proper development and survival in the host organism during infection. In this work, we present the metal-binding characteristics of the peptidic models of two putative Fe(II)-binding sites of *E. coli*FeoB: **L1** (Ac-_477_IMRGEATPFVMELPVYHVPH_496_–CONH_2_) being a fragment of the Core CFeoB region located between the transmembrane helices and **L2 (**Ac-_38_VERKEG_43_-CONH_2_), which represents the ExxE motif found within the NFeoB domain. With a variety of physicochemical methods, such as potentiometry, mass spectrometry, NMR, and EPR spectroscopy, we have determined the stability constants and metal-binding residues for the complexes of Fe(II), Mn(II), and Zn(II) with two ligands, **L1** and **L2**, acting as models for the Core CFeoB and ExxE motif. We compare their affinities toward the studied metal ions with the previously studied C-terminal part of the protein and discuss a possible role in metal trafficking by the whole protein.

## Abstract 2

Transmembrane FeoB protein is crucial for Fe(II) bacterial uptake. Despite its importance, protein’s metal-binding regions have not yet been elucidated. We have decided to investigate two putative Fe(II)-binding sites of FeoB: the Core CFeoB region and the ExxE motif found in the NFeoB domain. Herein, we present the results of the solution studies of Fe(II), Mn(II), and Zn(II) complexes formed with the peptidic models of the Core CFeoB region (**L1**) and the ExxE motif (**L2**).

## Introduction

Understanding of the coordination chemistry of proteins is often crucial for elucidating their function and mechanism of action. The binding of the metal ion can change the characteristics of the protein, its structure, activity, and ability to serve its function. Therefore, determining the metal-binding sites is vital for a thorough understanding of the nature of a specific protein. X- ray crystallography is a technique that provides precise information on the location of the metal-binding sites, the identity of the bound metal ions, and the coordinating ligands in the protein structure.^1^ However, to utilize it, a protein crystal of good quality must be obtained, which is not always feasible, especially for transmembrane proteins. Such proteins in their native state are anchored in the membrane, which determines their folding. In cases when a crystal of the protein cannot be obtained and characterized by X- ray crystallography, solution studies can be of help to characterize the coordination chemistry of the protein and determine the metal-binding sites. Since whole proteins are usually too large to be analyzed efficiently by solution studies, techniques such as potentiometric titrations and NMR spectroscopy of smaller peptidic fragments are often used to model fragments of the protein.^2−4^ In this approach, peptide sequences should be chosen carefully in order to contain the hypothetical metal-binding site. Next, the complexes formed between the peptide and the metal ions of interest can be characterized by a variety of techniques and yield information about the coordination chemistry of the peptide and, consequently, about the fragment of the protein modeled by the peptidic sequence. It must be noted, however, that the results obtained for peptidic models should be very carefully extrapolated to proteins, in which there are a plethora of interactions between regions and domains, lacking in the peptide studies.

We decided to use the approach described above to characterize the coordination chemistry of the fragments of the transmembrane FeoB protein, for which the crystal of the whole protein has not been obtained. While aware of the limitations of the approach and being careful with extrapolating the information obtained for the peptidic fragment to the protein fragment, we believe that this work is also an important input into the topic of the coordination chemistry of Fe(II) and Mn(II), which is significantly lacking in the literature, compared to metal ions like Cu(II) or Zn(II).

A bacterium’s ability to grow and survive in a host environment during an infection is strictly connected with its capacity to effectively take up indispensable metal ions from the host organism. This is mostly due to the metal ions being incorporated into a plethora of enzymes involved in crucial life processes, either in a catalytic or structural role.^5^ Divalent transition metal ions, such as Fe(II), Mn(II), and Zn(II), are essential for survival but can also be toxic in high concentrations;^6^ thus, their transport is tightly regulated by a whole array of systems, like (i) transmembrane protein transporters^7^ and ion channels^8^ responsible for import and export, (ii) low molecular weight, high-affinity chelators and metallophores,^9^ secreted by bacteria to scavenge metal ions from the environment, and (iii) specialized transcriptional regulatory proteins,^10,11^ to mention only the most important. Understanding the mechanisms operating in metal ion transporters can be significantly facilitated by coordination chemistry studies of the putative metal-binding regions of the transporter protein.^12^ That is especially true for transport systems for which elucidating the mechanism of action is still challenging. An example of such a system is the Feo system, which is regarded as the most important Fe(II)-specific bacterial transport system and seems to utilize the energy from GTP hydrolysis to transport the metal ion.^13−15^ The importance of the Feo transporter for bacteria is well reflected by hampered or completely reduced virulence in pathogenic bacteria with defective or deleted genes encoding the crucial transmembrane protein of the system, FeoB.^16−19^ Additionally, this reflects the crucial importance of Fe(II) acquisition in bacteria’s virulence, making it one of the most important virulence factors, especially for those pathogens occupying the anaerobic niches of the host, e.g., some parts of the mammalian digestive system.^20^

**Figure 1:**
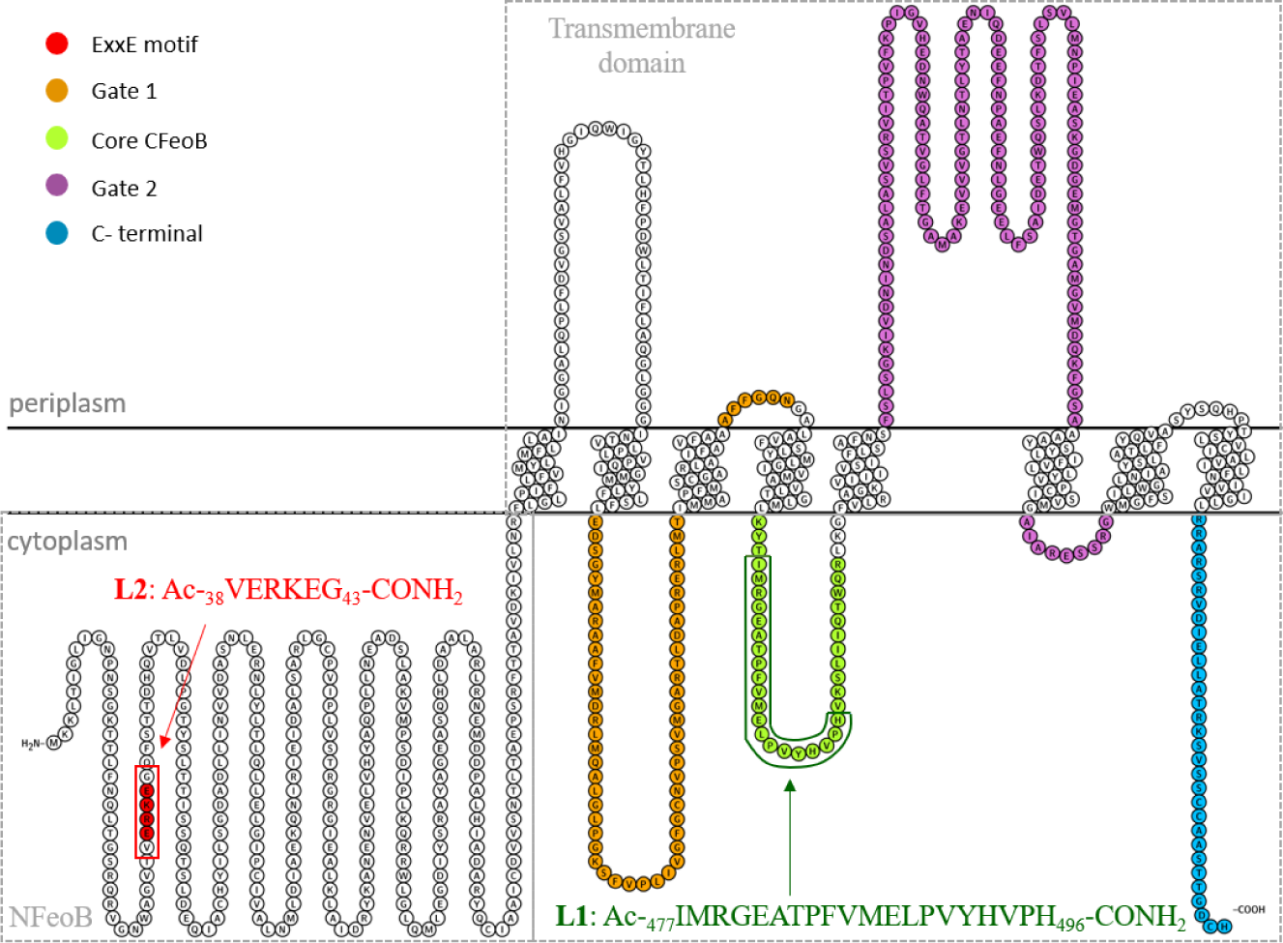
Predicted topology diagram of*E. coli*FeoB, with the periplasmic and cytoplasmic parts of putative Fe(II)-binding regions marked in colors. **L1** and **L2** sequences are indicated within the Core CFeoB region and the NFeoB ExxE motif, respectively, for which they act as a model. Topology prediction was carried out by DeepTMHMM server, visualized with the use of Protter tool.^25,26^ Reproduced from ref. (27). Copyright 2024 American Chemical Society.

Apart from the transmembrane FeoB, which is directly involved in the translocation of the Fe(II) ion across the inner membrane, some bacteria also possess the genes to encode two proteins of not yet established function, FeoA and FeoC.^13,14^ While there are some putative roles for FeoA and FeoC proposed in the literature, in this work, we decided to focus on FeoB. None of these proteins are located in the periplasm, as opposed to some other Fe(II) transport systems, *e.g*., YfeA in the YfeABCD system, FutA1 and FutA2 in FutABC, or EfeU and EfeO in EfeUOB, to name a few.^7,21,22^ This could mean that the transmembrane FeoB protein can efficiently attract and bind periplasmic Fe(II), without the need for auxiliary Fe(II)-binding proteins located in the periplasm. Hypothetical periplasmic metal-binding regions of the FeoB could be located within the recognized Gate motifs in the literature (Figure 1). These are two putative Fe(II)-binding sites, similar to the motifs present in the iron transporter found in yeast, Ftr1p, in which they function as channels for the metal ion.^14,23^ Another putative metal-binding region located in the transmembrane domain is the Core CFeoB of the protein, located between the Gate motifs (Figure 1), possibly cooperating with these motifs and facilitating the Fe(II) transport through the membrane.^24^ Unlike some other putative metal-binding regions, such as the cytoplasmic C-terminal part, which is well-conserved only in the Gammaproteobacteria class, the Core CFeoB region is present in all FeoB proteins, in both Gram-positive (G+) and Gram-negative (G−) bacteria, which could indicate its important biological role for protein function. Possessing conserved glutamic acid and histidine residues, the Core CFeoB region seems well-equipped for the hypothetical metal-binding role.

The FeoB protein can be divided into two main domains: the transmembrane domain described above, 492 amino acids long in *E. coli*, and the cytoplasmic NFeoB (N-terminal FeoB) domain, which consists of 281 amino acids. The NFeoB has been identified with GTP-binding motifs and has been shown to be able to bind and hydrolyze both GTP and ATP, which could then be used to translocate the metal ion in an active manner.^28,29^ While the crystal structures of the NFeoB domain are available, none of them involve the metal ions; thus, they do not shed light on the metal-binding properties of the domain. Apart from the suggested function of creating the driving force to transport Fe(II) across the inner membrane, the NFeoB domain also possesses a probable metal-binding sequence ExxE (glutamic acid-any amino acid-any amino acid-glutamic acid). Such a motif has been identified as a metal-binding sequence in a couple of iron-sensing or iron-transporting proteins in a variety of organisms, *e.g*., mammalian ferritin, *Saccharomyces* iron transporter FTR1, *C. albicans* iron permeases CaFTR1 and CaFTR2, periplasmic regions of PmrB proteins conserved in a variety of bacteria, such as *E. coli*, *Y. pestis*, *S. enterica*, *K. pneumoniae*, or bacterial EfeU transmembrane protein, homologous to yeast FTR1 transporter.^30−34^ The possibility of Fe(II) binding to the ExxE motif found in NFeoB was examined by Hung et al. by performing cleavage of the *E. coli* NFeoB with the use of Haber–Weiss reactions in the presence of Fe(II) and GTP, GDP, and GMPPNP nucleotides.^35^ The cleavage took place in the regions spatially near the ExxE motif, suggesting a possible Fe(II)-binding role. Mutations of glutamic acid residues to alanine, AxxA, resulted in phenotypes with moderate Fe(II) deficiency in genetic complementation tests. However, using Fenton reactions, these mutants also caused some cleavage to the protein, indicating that there might be some other residues important for iron binding and that further studies must be carried out to fully explore the possibility of Fe(II) binding by the ExxE motif.

**Figure 2:**
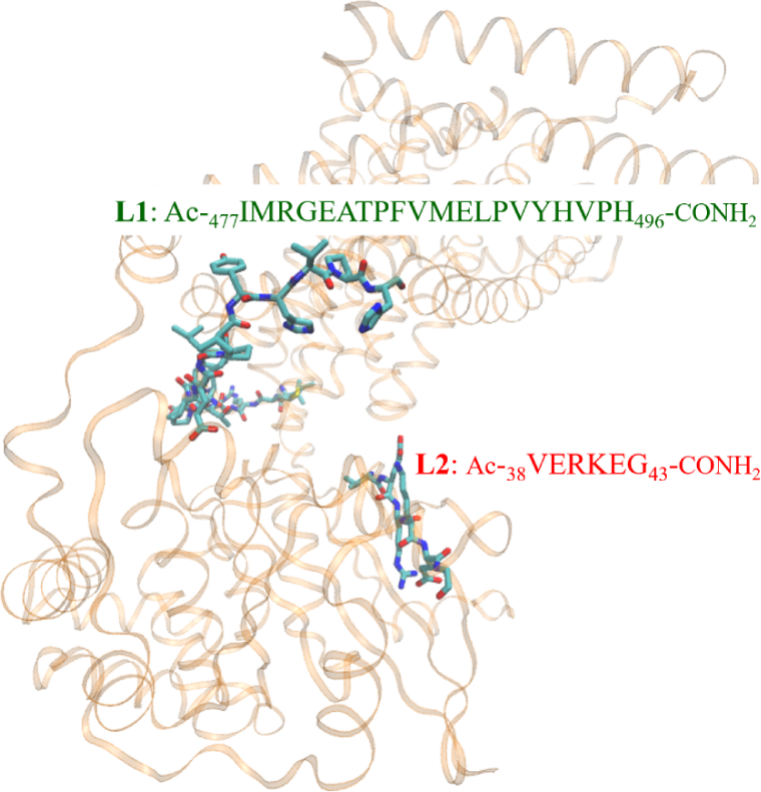
*E. coli* FeoB structure predicted by AlphaFold with **L1** and **L2** highlighted.^36,37^ Visualized with VMD 1.9.3 software.^38^ UniProt ID: P33650.

In our previous work, we studied the metal-binding properties of the peptidic models of the C-terminal cytoplasmic part of the*E. coli*FeoB protein (Figure 1) as the first part of a comprehensive characterization of the protein’s putative metal-binding regions.^27^ To further explore this subject, we decided to study the metal complexes of the peptide sequences from the K12*E. coli* Core CFeoB region and ExxE motif found in the NFeoB domain, which are proposed in the literature as hypothetical metal-binding sequences. Depicted in the structure of the whole protein in Figure 2, the chosen peptide sequences are as follows: Ac-_477_IMRGEATPFVMELPVYHVPH_496_–CONH_2_ (**L1**, Core CFeoB domain) and Ac-_38_VERKEG_43_-CONH_2_ (**L2**, NFeoB domain), depicted in Figures 1 and 2.

**Figure 3:**
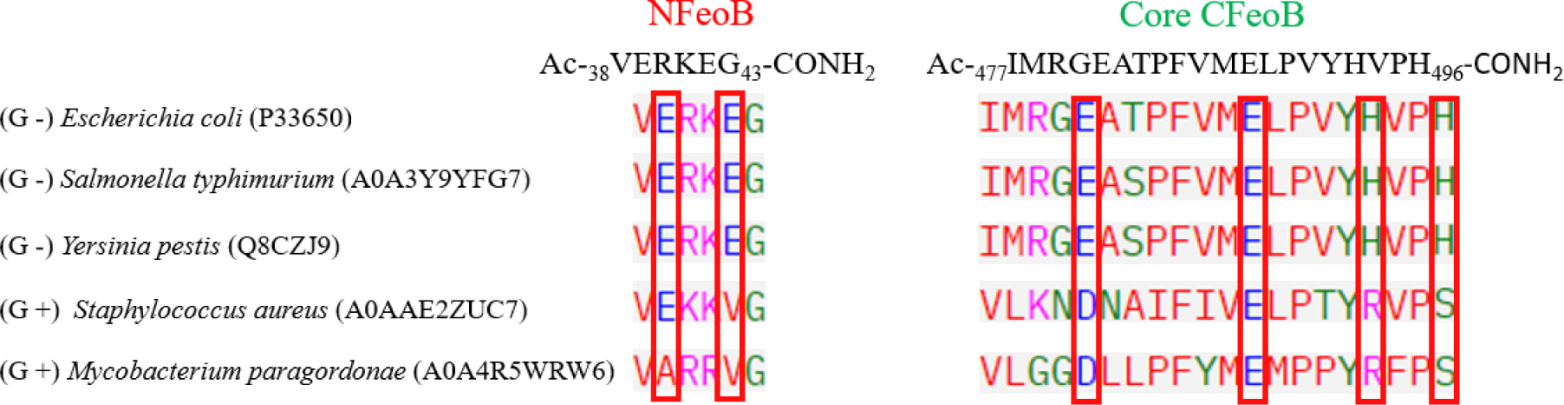
Sequence alignment of the chosen peptidic fragments in the sequence of FeoB protein from a variety of Gram-negative (*E. coli*, *S. typhimurium*, and *Y. pestis*) and Gram-positive (*S. aureus* and *M. paragordonae*) bacteria. Potential Fe(II)-binding ligands are marked with red rectangles. UniProt entry codes are listed in brackets. Alignment is carried out with Clustal Omega.^39^

The peptide sequences were chosen to include the conserved possible Fe(II)-binding residues, such as glutamic acids and histidines, to resemble the coordination properties of the Core CFeoB region and the ExxE motif (Figure 3). While the potential Fe (II)-binding ligands are well conserved in Gram-negative bacteria, this is not the case for Gram-positive bacteria shown in Figure 3, especially for the histidine residues in the Core CFeoB fragment and glutamic acid residues in the NFeoB fragment.

The N-terminus and C-terminus of the chosen peptides were acetylated and amidated, respectively, to resemble the native protein conditions. While the Feo system is believed to serve solely as an Fe(II) transporter, many of such transporters can also transport other divalent metal ions, mainly Mn(II) and Zn(II),^40^ therefore, we have decided to investigate also Mn(II) and Zn(II) complexes, which allowed us to explore the metal specificity of the chosen FeoB regions. Fe(II), Mn(II), and Zn(II) complexes with **L1** and **L2** were investigated with a variety of methods, such as mass spectrometry, potentiometric titrations, and nuclear magnetic resonance (NMR) and electron paramagnetic resonance (EPR) spectroscopies. From the collected data, we present the stoichiometry of the complexes, their stability constants, geometry, and propose metal-binding residues. We also present the binding affinities of **L1** and **L2** for each metal ion and compare it with those of the peptidic models of the C-terminal part of *E. coli* FeoB, which we have determined recently in a similarly carried-out research.^27^

## Experimental Section

### Materials

Studied ligands **L1** and **L2** were ordered from KareBay Biochem. Their identity was confirmed by ESI-MS experiments, and their purity was determined to be >98% by potentiometric measurements, using the Gran method. The metal ion solutions were prepared from the corresponding perchlorates (Sigma-Aldrich) in the case of Zn(II) and Mn(II). Fe(II) solutions were prepared anaerobically right before the experiments from Mohr’s salt (ammonium iron(II) sulfate, Sigma-Aldrich). Zn(II) and Mn(II) solutions were standardized by the ICP-OES method and complexometric titration with murexide and standardized ethylenediaminetetraacetic acid disodium salt (Na_2_H_2_EDTA). Fe(II) solutions were standardized by the colorimetric method using 1,10-phenanthroline (Sigma-Aldrich) under an argon atmosphere. Carbonate-free Titripur NaOH (Sigma-Aldrich) was standardized with potassium hydrogen phthalate (Sigma-Aldrich) and used as a titrant in the potentiometric experiments. Sodium perchlorate (Sigma-Aldrich) was used to adjust the ionic strength to *I* = 0.1 M, and perchloric acid (J.T. Baker) was used to adjust the pH of the samples. All samples were prepared by using double-distilled water. Because of the oxidation sensitivity of Fe(II), all samples containing Fe(II) were prepared in an argon atmosphere inside the glovebox, using deoxygenated solvents. Mohr’s salt was used as a stable Fe(II) source. All of the glass containing Fe(II) samples (potentiometric vessel, NMR tube, vial for mass spectrometry) was carefully sealed before being taken out of the glovebox for the experiments. In Fe(II) experiments, we did not observe the presence of Fe(III) ions. All Fe(II) samples were colorless throughout the experiments. Exposing the samples to air after finishing the experiments resulted in the formation of a yellow color as a result of the oxidation to Fe(III). The addition of the thiocyanate anions in an anaerobic atmosphere did not result in the formation of the red-colored Fe(III) complex in NMR samples after finished spectra acquisition. We did not observe any signals that could be assigned to Fe(III) in the mass spectra.

### Electrospray Ionization-Mass Spectrometry (ESI-MS)

All of the mass spectra were acquired on a Bruker Q-TOF compact spectrometer. The appropriate amounts of Fe(II), Mn(II), and Zn(II) solutions were added to the stock **L1** and **L2** solutions (C = 1 × 10^–4^ M) to obtain a 1:1 and 1:2 (M:L) ratio. All of the samples were prepared in a 50:50 (w/w) methanol/water solvent and diluted with methanol before injection into the spectrometer. The spectra were acquired in the positive ion mode. The TuneMix mixture (Bruker Daltonics) was used to calibrate the instrument. Some of the instrumental parameters used were as follows: scan range, *m*/*z* = 200–2000; dry gas, nitrogen; *T* = 170 °C; capillary voltage, 4500 V; ion energy, 5 eV. The data were processed with the use of the Compass Data Analysis 4.0 software (Bruker Daltonics). All of the solvents used were of liquid chromatography–mass spectrometry grade.

### Potentiometric Titrations

Potentiometric titrations were carried out on a Metrohm Titrando 905 titrator connected to the Metrohm Dosino 800 dosing system. The potentiometric cell glass was equipped with the microdelivery buret tube, magnetic stirrer, and an inlet–outlet tube for argon gas. An InLab Semi-Micro pH electrode (Mettler-Toledo) was used as a pH sensor. The electrode was calibrated daily for the hydrogen ion concentration by titrating 2 mL of 4 mM perchloric acid with sodium hydroxide. The stability constants of the proton, Fe(II), Mn(II), and Zn(II) complexes with ligands were determined using the titration curves from pH 2 to 11 at a temperature of 298 K, with the use of SUPERQUAD^41^ and HYPERQUAD 2008^42^ software. The samples used for titrations contained the ligand at a concentration of 0.5 mM, perchloric acid at a concentration of 4 mM, and 0.1 M sodium perchlorate as an ionic strength. The exact concentrations of the ligand solutions were determined by the Gran method. The solutions of Fe(II), Mn(II), and Zn(II) were prepared from Mohr’s salt, manganese perchlorate, and zinc perchlorate, respectively, and added to the sample solution to achieve a 1:1.1 Fe(II):L and Zn(II):L ratio and for Mn(II):L, a 1:1.1 and 1:2 ratio. All titrations were performed under an argon atmosphere using a carbonate-free, standardized sodium hydroxide base as a titrant. Two titrations were carried out for the ligands, as well as for each of the ligand-metal ion systems. Standard deviations were calculated by HYPERQUAD 2008 and refer to random errors only. HYSS software was used to create competition and speciation diagrams and calculate pM and *K*_*d*_ values.^43^ Fe(II), Mn(II), and Zn(II) hydrolysis constants were taken into account for the calculations of stability constants of complexes (Table S1).^44,45^

### Nuclear Magnetic Resonance (NMR) Spectroscopy

NMR experiments were performed using a Bruker Ascend 400 MHz spectrometer equipped with a 5 mm automated tuning and matching broadband probe (BBFO) with z-gradients. Samples utilized for NMR experiments ranged from 0.4 to 1.0 mM and were dissolved in a 90/10 (v/v) H_2_O–D_2_O solvent mixture. All NMR experiments were performed at 298 K in 5 mm NMR tubes. The 2D ^1^H–^13^C heteronuclear correlation spectra (HSQC) were acquired using a phase-sensitive sequence employing Echo-Antiecho-TPPI gradient selection with a heteronuclear coupling constant of JXH = 145 Hz and shaped pulses for all 180° pulses on the f2 channel with decoupling during acquisition. Sensitivity improvement and gradients in back-INEPT were also employed. Relaxation delays of 2 s and 90° pulses of about 10 μs were applied for all experiments. Solvent suppression was achieved using excitation sculpting with gradients. The spin-lock mixing time of the ^1^H–^1^H TOCSY experiment was obtained with MLEV17. ^1^H–^1^H TOCSY experiments were performed using a mixing time of 60 ms. ^1^H–^1^H ROESY spectra were acquired with spin-lock pulse durations in the range of 200–250 ms. The assignments of ^1^H and ^13^C were made by a combination of mono- and bidimensional and multinuclear NMR techniques: ^1^H–^1^H TOCSY, ^1^H–^13^C HSQC, and ^1^H–^1^H ROESY, at different pH values. To avoid severe broadening of the signals because of the paramagnetic character of Mn(II) and Fe(II), the NMR experiments were performed with the subsequent addition of a substoichiometric amount of metal ion to the ligand solution. All NMR data were processed using TopSpin (Bruker Instruments) software and analyzed using Sparky 3.11 and MestReNova 6.0.2 (Mestrelab Research S.L.) programs.

### Electron Paramagnetic Resonance (EPR) Spectroscopy

EPR spectra were recorded using a Bruker ELEXSYS E500 CW-EPR spectrometer equipped with an NMR teslameter (ER 036TM) and a frequency counter (E 41 FC) at X-band frequency, at room temperature. The peptide concentration was 0.5 mM, and the metal: ligand molar ratio was 1:1.1. EPR parameters were obtained by using the Bruker WinEPR SimFonia program and Doublet new (EPR of S = 1/2) program by A. Ozarowski (National High Field Magnetic Laboratory, University of Florida, Gainesville, FL).

### UV–Vis Spectroscopy

The absorption spectra were recorded under an inert atmosphere using a Jasco V-730 UV–visible spectrophotometer in the 350–650 nm range, using a quartz cuvette with a 0.1 cm optical path, scanning speed: 400 nm/min, data pitch: 0.5 nm, number of accumulations: 1. The colorimetric Fe(II) concentration determination utilized the formation of a 1:3 M:L complex of Fe(II) with 1,10-phenanthroline, with λ_max_ = 510 nm. First, the calibration curve was prepared for the Fe(II) ion concentration in the range of 0.1–1.1 mM, and a linear function correlating the absorption of the solution with the Fe(II) concentration was obtained. Then, the calibration curve was used to determine the concentration of the freshly prepared Fe(II) stock solution by measuring the absorption at 510 nm of the three samples made from the stock solution and taking the average of the concentration calculated for each sample. The ratio of Fe(II) to 1,10-phenanthroline was 1:5 to ensure complete complexation of the metal ion.

## Results and Discussion

The complexes of both ligands, **L1** (Ac- I_1_M_2_R_3_G_4_E_5_A_6_T_7_P_8_F_9_V_10_M_11_E_12_L_13_P_14_V_15_Y_16_H_17_V_18_P_19_H_20_-CONH_2_) and **L2 (**Ac–V_1_E_2_R_3_K_4_E_5_G_6_-CONH_2_) with Fe(II), Mn(II), and Zn(II) ions were investigated using a variety of physicochemical methods. ESI-MS experiments revealed the stoichiometry of the complexes formed in the studied systems. The data obtained through potentiometric titrations enabled us to determine the protonation constants of the ligands and the stability constants of the metal complexes and to draw the speciation plots for each system. *K*_*d*_ and pM values were also calculated from the potentiometric data. NMR and EPR spectroscopy provided insights into the coordination of the metal ion and the geometry of the complexes.

**L1** possesses five groups able to deprotonate in the studied pH range (2–11). Utilizing potentiometric titrations, we determined the p*K*_a_ values of the deprotonating groups (Figure S1a and Table 1). Two glutamic acid residues (E_5_ and E_12_) are the first to deprotonate, with the p*K*_a_ values of their side-chain carboxylic groups being 4.15 and 4.91. The next two dissociation constants can be attributed to the deprotonation of the imidazole ring of the histidine residues (H_17_ and H_20_). As in the case of the glutamic acid residues, we cannot assign the p*K*_a_ values (6.26 and 6.98) to the specific histidine residue. The last dissociation constant arises from the deprotonation of the tyrosine’s (Y_16_) hydroxyl group (p*K*_a_ = 9.67). The dissociation constant of the arginine’s guanidine group could not be determined by the potentiometric titrations as the deprotonation most probably occurs at pH values well above the studied range. Therefore, arginine’s side chain remains positively charged throughout the studied pH range. The charge of the completely deprotonated [L] form is −2 and −1 for **L1** and **L2**, respectively. The determined p*K*_*a*_ values are consistent with those reported in the literature.^46^

**Table 1:** Protonation Constants (logβ) and p*K*_a_ Values of the Ligands **L1** and **L2**^a^

Peptide	Species	logβ^b^	p*K*_a_^c^	Deprotonating residue
**L1**	[H_5_L]^3+^	31.97(5)	4.15	Glu
	[H_4_L]^2+^	27.82(5)	4.91	Glu
	[H_3_L]^+^	22.91(4)	6.26	His
	[H_2_L]	16.65(3)	6.98	His
	[HL]^−^	9.67(2)	9.67	Tyr
**L2**	[H_3_L]^2+^	17.95(6)	3.59	Glu
	[H_2_L]^+^	14.36(7)	4.44	Glu
	[HL]	9.92(3)	9.92	Lys

a *T* = 298 K, *I* = 0.1 M NaClO_4_, standard deviations on the last digit given in parentheses.

b Overall stability constants (β) expressed by the equation: β(H_n_L) = [H_n_L]/[L][H+]^n^.

c Acid dissociation constants (p*K*_a_) expressed as p*K*_a_ = logβ(H_n_L) – logβ(H_n–1_L).

**L2** is significantly shorter than **L1**, being only 6 amino acids long. In the studied pH range, it behaves like an H_3_L acid, exhibiting three dissociation constants (Figure S1b, Table 1). The first two can be attributed to the side-chain carboxylic groups of the glutamic acid residues, E_2_ and E_5_, with p*K*_a_ values of 3.59 and 4.44. From the potentiometric data, it is not possible to assign the dissociation constants to specific glutamic acid residues. It is worth noting that the p*K*_a_ value of 3.59 is quite low for a glutamic acid residue. We believe it is a consequence of the positively charged arginine (R_3_) and lysine (K_4_), which are protonated at acidic pH and create an overall positive charge on the relatively small peptide. This most probably influences the early deprotonation of the glutamic acid residue and creates a local negative charge that can interact with positively charged R_3_ and K_4_. The last constant can be attributed to the deprotonation of the lysine (K_4_) residue (p*K*_a_ = 9.92). Similarly, for **L1**, we could not determine the p*K*_a_ of the arginine residue. The protonation constants of the ligands are listed in Table 1.

### Stoichiometry of Metal Complexes

The stoichiometry of the complexes formed between Fe(II), Zn(II), and Mn(II) and the studied ligands **L1** and **L2** was investigated by the ESI-MS method. For all studied systems, we found that only 1:1 (M:L) complexes were formed. The correct peak assignment was ensured by comparing the isotopic distributions of the simulated and experimental spectra. The comparison of the simulated and experimental *m*/*z* values for the most abundant ligand and complex signals in the spectra is collected in Table S2. Mass spectra of the studied systems are presented in Figures S2–S7.

### Iron Complexes

**Figure 4:**
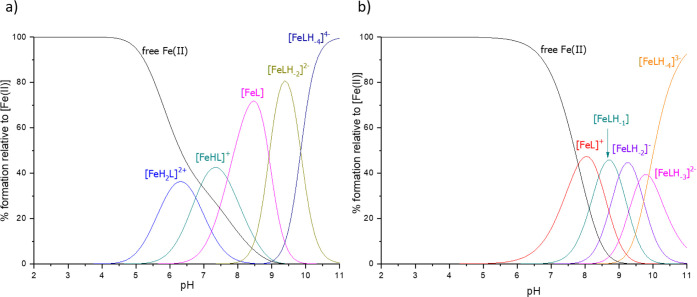
Distribution diagrams of complexes formed between Fe(II) and ligands: (a) **L1** and (b) **L2**. Species distributions calculated for NMR experimental conditions: [L]_tot_ = 0.5 mM; Fe(II):L = 1:3.

In the Fe(II):**L1** system, the complexation starts at a pH of about 4.0, with the formation of [FeH_2_L]^2+^ species (Figure 4a). Both of the glutamic acid residues (E_5_ and E_12_) and one histidine residue (H_17_ or H_20_) are deprotonated in this form. The NMR spectra recorded at pH = 5.5 showed minimal changes compared to the spectra of the free ligand (Figure S8). This could be due to the low abundance of the complexed iron (over 80% of Fe(II) in the solution remains in a free form), as indicated in the species distribution diagram (Figure 4a). The next form, [FeHL]^+^, dominates in the solution in the pH range of about 6.8–7.7 and contains another histidine residue in a deprotonated form. The difference between its p*K*_a_ value in the complex (6.76) and the free ligand (6.98, Table 1) is quite low, suggesting weak or no iron binding. However, in the NMR spectra recorded at pH = 7.7, in which in the solution there is a mixture of [FeHL]^+^ and [FeL] forms, the signals attributed to histidine residues (H_17_ and H_20_) are noticeably perturbed, indicating their likely involvement in metal ion binding in a {2 N_im_} mode. This inference is supported by the selective disappearance of signals from these two residues and significant broadening of signals from neighboring residues, including tyrosine Y_16_, valine V_18_, and proline P_19_ (Figure S3). No trace of involvement of glutamic acid residues was observed in the spectra, which could explain the complexation process starting only after the first histidine begins to deprotonate at a pH of about 4.0. From a pH of around 7.7, a new form starts to dominate in the solution: [FeL]. The p*K*_a_ value of the deprotonation from [FeHL]^+^ to [FeL] form is 7.69. We believe that this value is too low to represent the deprotonation of the tyrosine’s hydroxyl group, which most probably stays as a nonbinding residue in the studied system. The observation is supported by NMR spectra recorded at pH = 7.7, where in both the free ligand and Fe(II) system, Y_16_ is still protonated (Figure S9). Thus, the deprotonation step has to be attributed either to a water molecule from the Fe(II) coordination sphere or to an amide group from the peptide bond. The possibility of Fe(II) binding by the amide groups has been proposed in the literature,^47−52^ as well as in our previous work on similar systems,^27^ in which the p*K*_a_ values were in the range of 8.73–10.44. Another two deprotonations result in the formation of the [FeLH_–2_]^2–^ species. The confirmation of Fe(II) binding by H_17_ and H_20_ for this species is further substantiated by the NMR spectra recorded at pH = 9.4, in which distinct disappearances of imidazolic protons Hδ1 and Hε2 are indicative of the interaction between these histidine residues and the Fe(II) ion. Because we could not determine the stability constant for [FeLH_–1_]^−^, most probably being just a transient form with a very low concentration in the solution, we cannot determine the specific p*K*_a_ values for the two subsequent deprotonations. However, with the average p*K*_a_ of 8.92, we believe that these correspond to other amide groups or water molecules. The stability constant of the complex form [FeLH_–3_]^3–^ could not be determined, most likely due to the same reason as that of [FeLH_–1_]^−^. The last species formed in the solution is [FeLH_–4_]^4–^, a result of subsequent deprotonation of most probably another amide group or water molecule and nonbinding tyrosine residue (average p*K*_a_ = 9.86).

For the Fe(II):**L2** system, five complex species could be detected by potentiometry (Figure 4b). The complexation starts only at a pH above 5, in the [FeL]^+^ form, with both of the glutamic acid residues deprotonated and most probably coordinating the metal ion in {2 COO^–^} mode. The complexation process is rather weak as at pH = 6.5, still about 95% of Fe(II) in the solution exists in the free form. NMR studies conducted at pH 5.2 indicate that the Fe(II):**L2** system remains similar to the free **L2** system, consistent with the very low concentration of species formed at this pH. However, as the pH increases to 8.2, perturbations are observed in both E_2_ and E_5_ signals, as well as neighboring residues (such as R_3_ and K_4_), which experience severe line broadening due to their proximity to the Fe(II) ion bound to the carboxylic moieties of glutamate residues (Figure S10). The next forms, [FeLH_–1_] to [FeLH_–3_]^2–^, most probably result from the amide group or water molecule deprotonation (Table 2). The following and last complex species formed, [FeLH_–4_]^3^, is probably a consequence of the nonbinding lysine residue deprotonation, with the p*K*_a_ value (9.87) similar to the one in the free ligand (9.92). The stability constants for Fe(II):peptide systems are collected in Table 2.

**Table 2:** Stability Constants (logβ) and p*K*_a_ Values of the Fe(II):Peptide Systems^a^

Peptide	Species	logβ^b^	p*K*_a_^c^	Deprotonating residue
**L1**	[FeH_2_L]^2+^	20.22(9)	6.76	His
	[FeHL]^+^	13.46(8)	7.69	N_amide_ or O_water_
	[FeL]	5.77(5)	-	2 × (N_amide_ or O_water_)
	[FeLH_–2_]^2–^	–12.06(5)	-	N_amide_ or O_water_ and Tyr
	[FeLH_–4_]^4–^	–31.77(5)	-	-
**L2**	[FeL]^+^	5.52(10)	8.41	N_amide_ or O_water_
	[FeLH_–1_]	–2.89(4)	8.99	N_amide_ or O_water_
	[FeLH_–2_]^−^	–11.88(7)	9.57	N_amide_ or O_water_
	[FeLH_–3_]^2–^	–21.45(5)	9.87	Lys
	[FeLH_–4_]^3–^	–31.32(5)	-	-

a *T* = 298 K, *I* = 0.1 M NaClO_4_, standard deviations given in parentheses.

b Overall stability constants (β) expressed by the equation: β(Fe(II)H_n_L) = [Fe(II)H_n_L]/([Fe(II)][[L][H^+^]^n^).

c Acid dissociation constants (p*K*_a_) expressed as p*K*_a_ = logβ(Fe(II)H_n_L) – logβ(Fe(II)H_n–1_L).

### Manganese Complexes

**Figure 5:**
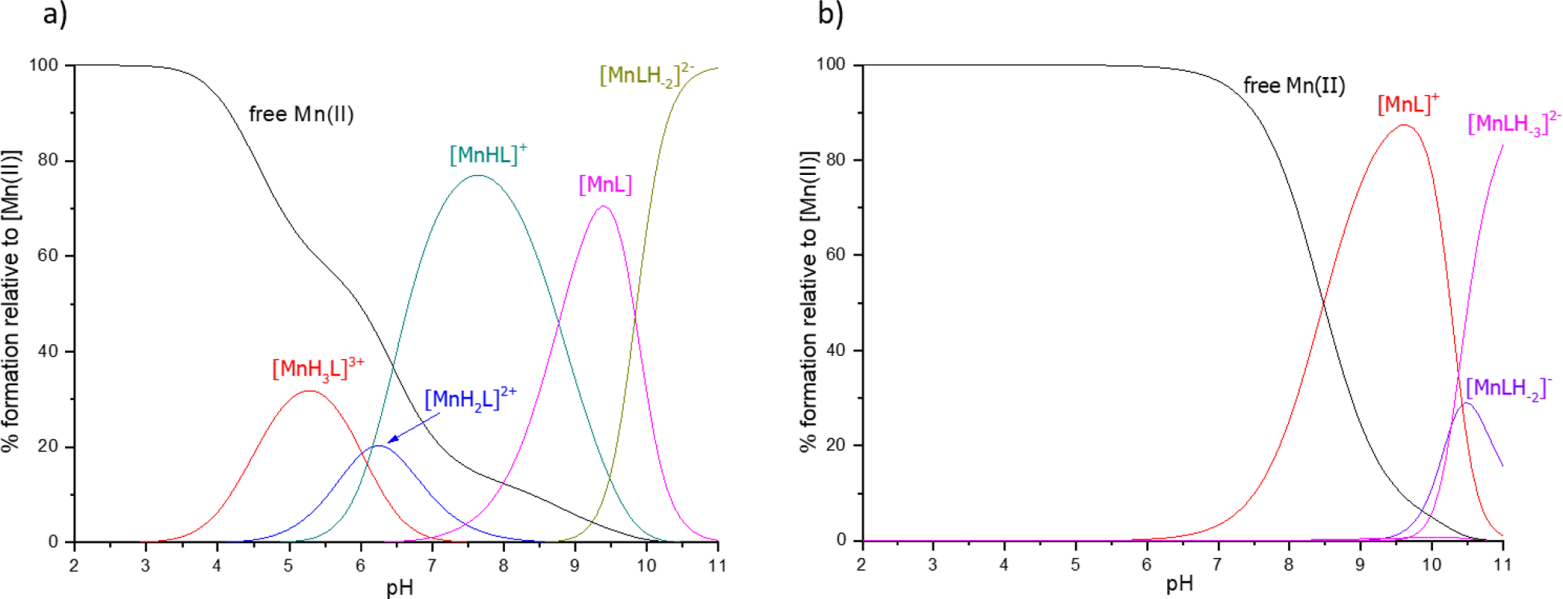
Distribution diagrams of complexes formed between Mn(II) and ligands: (a) **L1** and (b) **L2**. Species distribution calculated for NMR experimental conditions: [L]_tot_ = 0.5 mM; Mn(II):L = 1:50.

**Figure 6:**
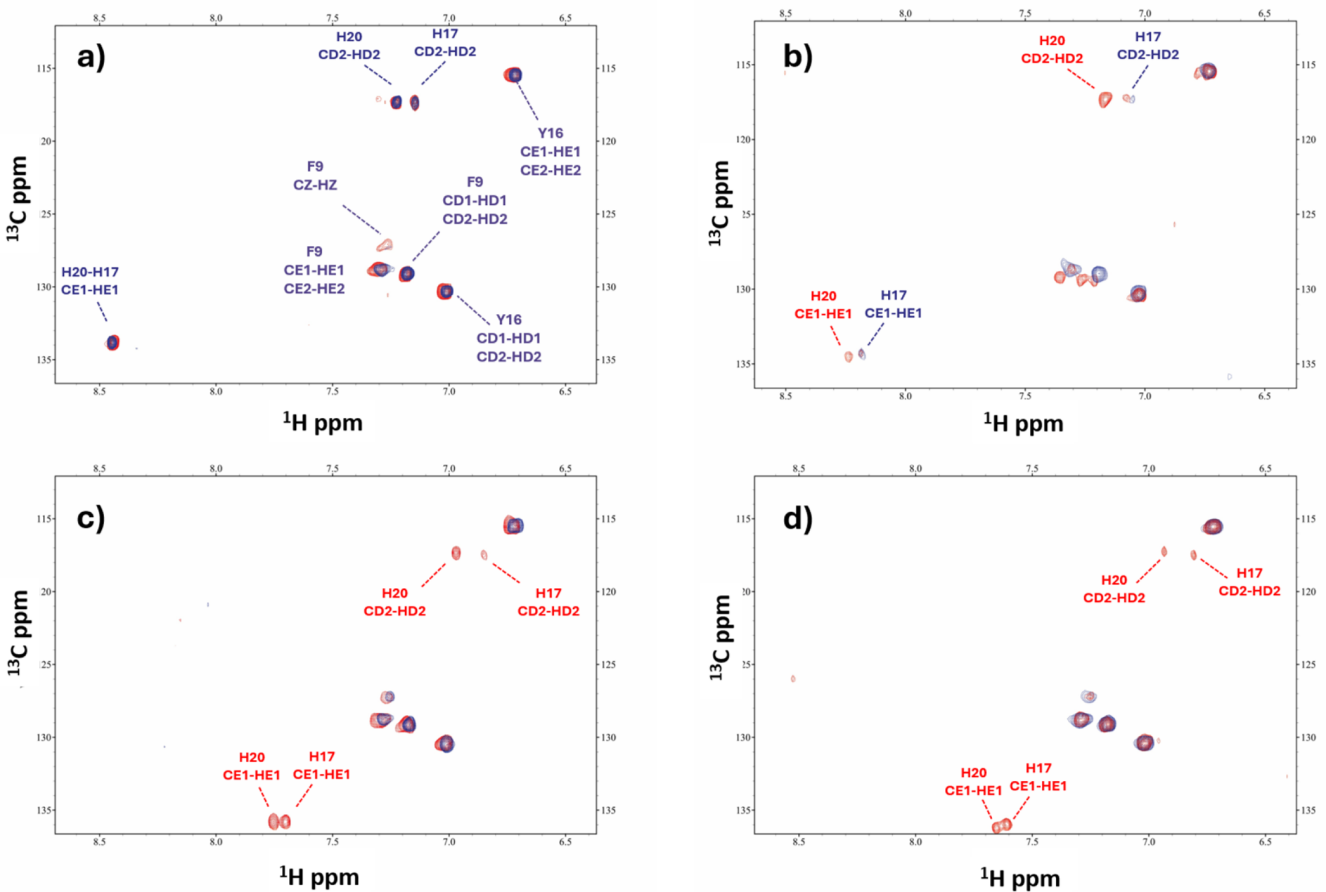
Comparison of the aromatic region in the ^1^H–^13^C HSQC spectra between the free peptide **L1** (red) and the Mn(II):**L1** system (blue) at a 1:50 molar ratio across various pH conditions: (a) pH = 4.9, (b) pH = 6.0, (c) pH = 7.0, and (d) pH = 9.0. Perturbed signals are indicated in red.

The complexation in the Mn(II):**L1** system starts at a pH of about 3.0, with the [MnH_3_L]^3+^ species (Figure 5a). This form contains both of the glutamic acid residues in a deprotonated form, most likely coordinating the metal ion in the {2 COO^–^} mode. This aligns with the observations from the NMR spectra, where the subtle changes in the E_5_ and E_12_ signals, initially noted at pH = 3.8, become more pronounced as the pH increases to 4.9. Under these pH conditions, histidines are not involved in metal complexation (Figure 6a). The next two forms, [MnH_2_L]^2+^ and [MnHL]^+^, arise from the deprotonations of the two histidine residues, with p*K*_a_ values of 6.04 and 6.15, respectively. These values are about 0.15 and 0.8 lower than the respective ones in the free ligand, suggesting a rather weak involvement of the first histidine residue in metal binding and a stronger binding of the second one. This is reflected in the species distribution plot (Figure 5a), in which [MnH_2_L]^2+^ shows low abundance, with [MnHL]^+^ quickly starting to dominate. The NMR spectra acquired at pH = 6.0, alongside observations of the shifts of both E_5_ and E_12_ signals, notably demonstrate the disappearance of the imidazolic signals associated with histidine H_20_, while H_17_ exhibits comparatively lesser perturbation (Figure 6b). Therefore, in the [MnHL]^+^ species, it is highly likely that the metal ion is bound to the peptide in a {2 COO^–^, 2 N_im_} coordination mode involving two carboxyl and two imidazole groups, consistent with observations from NMR spectra acquired at pH = 7.0 and 7.8 (Figures 6c, S11 and S12). The next deprotonation can most likely be attributed to a water molecule deprotonation (p*K*_a_ = 8.76) and result in the formation of [MnL] species. Tyrosine is most probably not involved in the metal binding as the signals related to Y_16_ are not affected in the NMR spectra at pH = 9.0 (Figure 6d). The last complex form detected in the solution is [MnLH_–2_]^2–^ as [MnLH_–1_]^−^ is probably just a transient form, for which the stability constant could not be determined precisely. The two successive deprotonations observed from [MnL] to [MnLH_–2_]^2–^ are again most likely associated with the deprotonation of another water molecule and the noncoordinating tyrosine residue (with an average p*K*_a_ of 9.83), as corroborated by NMR spectra acquired at pH = 10.4 (Figure S13).

For the Mn(II):**L2** system, the first complex species calculated in the speciation model is [MnL]^+^ (Figure 5b). Starting to form close to pH = 6.0, this species contains both of the glutamic acid residues deprotonated. The NMR titration for the Mn(II):**L2** system was conducted at pH = 5.1, with subsequent substoichiometric additions of the paramagnetic metal ion to the **L2** solution. The signals exhibiting selective broadening were primarily those associated with the glutamate residues (Figures S14 and S15). At pH = 7.0, we observe a replication of the aforementioned behavior (Figure S16). The spectra exhibit remarkable consistency with regard to the coordination of E_5_ and E_12_ with Mn(II). As in the NMR spectra, we can observe the complexation process happening at a pH a bit lower than in the speciation plot (Figure 5b). It is likely that the form [MnHL]^2+^ is forming in the solution at a pH of around 5; however, its stability constant could not be calculated in the proposed model with an adequate standard deviation (3σ value), probably due to the low concentration in the solution. However, as Mn(II) is paramagnetic, even small interactions in the solution could be pronounced as a broadening of the signals in the NMR spectra, which could explain the slight difference between the speciation plot (Figure 5b) and the NMR spectra acquired at pH = 5.1.

As most likely transient [MnLH_–1_] species could not be detected, the next form is [MnLH_–2_]^−^, with the two subsequent deprotonations (with the average p*K*_a_ = 10.42) that can be ascribed to the nonbinding lysine residue and a water molecule or two water molecules. Possibly, another water molecule deprotonation or lysine deprotonation (p*K*_a_ = 10.27) leads to the [MnLH_–3_]^2–^ form. As in the Fe(II) system, the metal ion binding by the ligand is achieved only through the two glutamic acid residues in all species. The stability constants determined for Mn(II):peptide complexes are collected in Table 3.

**Table 3:** Stability Constants (logβ) and p*K*_a_ Values of the Mn(II):Peptide Systems^a^

Peptide	Species	logβ^b^	p*K*_a_^c^	Deprotonating residue
**L1**	[MnH_3_L]^3+^	26.17(9)	6.04	His
	[MnH_2_L]^2+^	20.13(9)	6.15	His
	[MnHL]^+^	13.98(4)	8.76	O_water_
	[MnL]	5.22(4)	-	Tyr and O_water_
	[MnLH_–2_]^2–^	–14.45(3)	-	-
**L2**	[MnL]^+^	4.76(9)	-	Lys and O_water_ or 2 × O_water_
	[MnLH_–2_]^−^	–16.08(7)	10.27	Lys or O_water_
	[MnLH_–3_]^2–^	–26.35(8)	-	-

a *T* = 298 K, *I* = 0.1 M NaClO_4_, standard deviations (3σ values) are given in parentheses.

b Overall stability constants (β) expressed by the equation: β(Mn(II)H_n_L) = [Mn(II)H_n_L]/([Mn(II)][[L][H^+^]^n^).

c Acid dissociation constants (p*K*_a_) expressed as p*K*_a_ = logβ(Mn(II)H_n_L) – logβ(Mn(II)H_n–1_L).

The EPR spectra were recorded for Mn(II):**L1** and Mn(II):**L2** systems at various pH conditions, at room temperature. All of the spectra exhibit a distinctive six-line pattern, as a result of Mn(II) nuclear spin, , due to the hyperfine splitting of the allowed transitions. For all recorded spectra, the g-factor value is around 2.0, and the hyperfine coupling constant, A, is about 95 G. This is consistent with the octahedral hexaaqua Mn(II) complex.^53,54^ The intensity of the spectra decreases with rising pH as the complexation of manganese by the peptide ligands takes place. Due to the zero-field splitting, caused by the change in the ligand field of the metal ions, the broadening of signals of Mn(II) bound to studied ligands prevents signal detection.^55^ Thus, the decreasing intensity of the Mn(II) signal in EPR spectra reflects the complexation of the metal ion by **L1** and **L2**. The spectra are collected in Figure S17.

### Zinc Complexes

**Figure 7:**
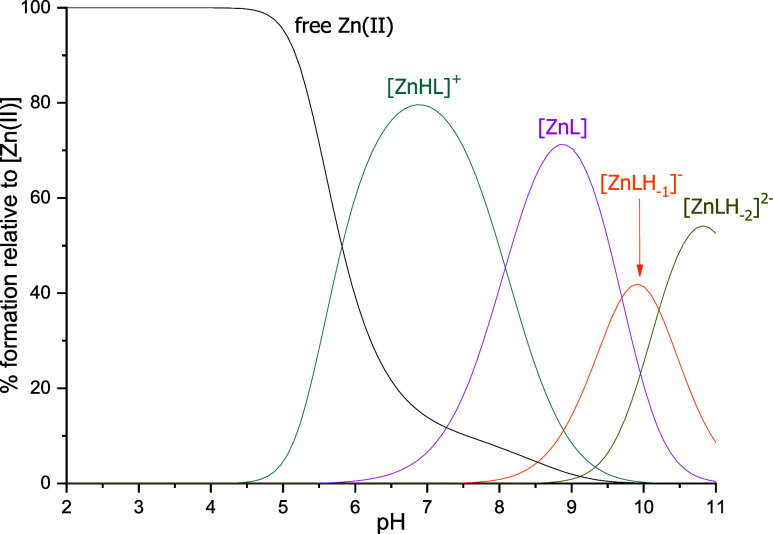
Distribution diagrams of complexes formed between Zn(II) and **L1**. Species distribution calculated for NMR experimental conditions: [Zn(II)]_tot_= 0.5 mM; Zn(II):L = 1:1.

**Figure 8:**
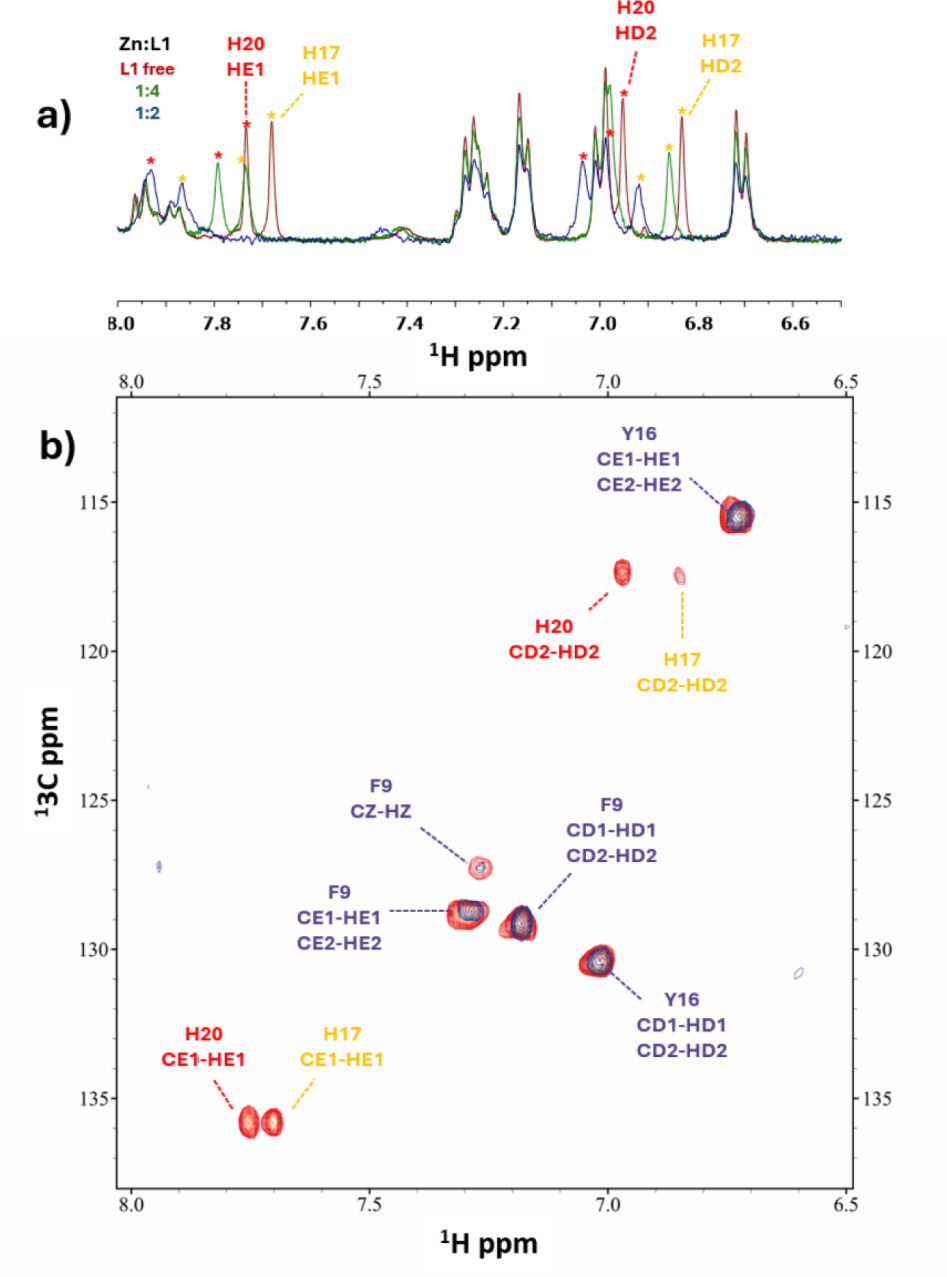
(a) Comparison of ^1^H spectra and (b) ^1^H–^13^C HSQC spectra in the aromatic region for the free peptide **L1** (red) with those following the sequential addition of Zn(II) at pH = 7.0. Perturbed signals of H_20_ are indicated in red and those of H_17_ in orange.

The first complex form in the Zn(II):**L1** system is [ZnHL]^+^ present in the solution from a pH of about 4.5 (Figure 7). While both of the glutamic acid residues and both of the histidine residues are already deprotonated in this complex form, we believe that the coordination of the metal ion is achieved only through the imidazole ring of the histidine residues {2 N_im_}, similarly to Fe(II). This is reflected in the NMR spectra recorded at pH = 5.4 (Figure S18), where an initial perturbation of the aromatic signals associated with the imidazolic nuclei of H_17_ and H_20_ becomes noticeable, a phenomenon that intensifies as the pH increases to 7 (Figure 8). No significant changes were observed in the E_5_ and E_12_ signals compared to the spectra of the free ligand under both pH conditions.

The next species forming in the solution is [ZnL], with a p*K*_a_ value of 8.08. This can most likely be attributed to a water molecule deprotonation. The last two forms, [ZnLH_–1_]^−^ and [ZnLH_–2_]^2–^, are most likely a result of the deprotonation of the nonbinding tyrosine residue and another water molecule. The respective p*K*_a_ values attributed to the deprotonations leading to the formation of [ZnLH_–1_]^−^ and [ZnLH_–2_]^2–^ are 9.70 and 10.21. The values of the stability constants for the Zn(II):**L1** system are collected in Table 4.

**Table 4:** Stability Constants (logβ) and p*K*_a_ Values of the Zn(II):Peptide Systems^a^

Peptide	Species	logβ^b^	p*K*_a_^c^	Deprotonating residue
**L1**	[ZnHL]^+^	15.04(2)	8.08	O_water_
	[ZnL]	6.96(6)	9.70	Tyr or O_water_
	[ZnLH_–1_]^−^	–2.74(6)	10.21	Tyr or O_water_
	[ZnLH_–2_]^2–^	–12.95(8)	-	-

a *T* = 298 K, *I* = 0.1 M NaClO_4_, standard deviations are given in parentheses.

b Overall stability constants (β) expressed by the equation: β(Zn(II)H_n_L) = [Zn(II)H_n_L]/([Zn(II)][[L][H^+^]^n^).

c Acid dissociation constants (p*K*_a_) expressed as p*K*_a_ = logβ(Zn(II)H_n_L) – logβ(Zn(II)H_n–1_L).

For the Zn(II):**L2** system, we had trouble determining the stability constants of the complexes. In potentiometric titrations, we could observe a precipitation starting at a pH of about 9.0, which most probably was solid Zn(OH)_2_. As the complexation process was starting at a rather high pH, it was not possible to determine a reliable complexation model with accurate values of stability constants. The same issues with precipitation were observed in the NMR experiments, which, however, provided us with some insights into the formed complexes. The NMR spectra obtained at pH = 5.1 and pH = 7.0, using a 1:1 (metal to ligand) molar ratio, exhibited spectral patterns similar to those of the free ligand. This resemblance suggests minimal interaction or perturbation between the metal and ligand under these conditions. A more careful analysis of the spectra revealed a new set of signals associated with the zinc-bound system, coexisting at a low concentration with free peptide signals, consistent with a slow-exchange regime where both species are distinct. At a 2:1 (metal to ligand) molar ratio and pH = 7.0, the set of signal shifts observed are consistent with those seen at a 1:1 ratio, but they appear slightly more pronounced (Figure S19). The chemical shift differences between the metal-bound peptide and the free peptide have been plotted in a diagram shown in Figure S20. Based on the ESI-MS experiments, it has been demonstrated that only 1:1 (metal to ligand) complexes are formed. Therefore, we infer that the shifts observed in the signals at higher metal/ligand ratios are not indicative of 2:1 complex formation. Instead, these shifts suggest that the complexes are relatively unstable, requiring additional Zn(II) ions to effectively facilitate the complexation process. Notably, the shifts in the backbone nuclei for E_2_, R_3_, K_4_, and E_5_ suggest a rearrangement of the peptide backbone, indicating a likely involvement of E_2_ and E_5_ in zinc coordination, which in turn induces the folding of the peptide to enable both residues to coordinate with the metal ion. However, in this scenario, we would have expected more pronounced alterations in the chemical shifts of the side chain of Glu residues (specifically β and γ nuclei proximal to the carboxyl moiety) upon binding to the metal ion. This behavior is similarly observed in the NMR spectra recorded at pH = 9.0; however, precipitation prevented us from obtaining a more detailed view of the system at this higher pH. While for Fe(II) and Mn(II) binding by **L2** was very weak, only for Zn(II) could we determine a reliable potentiometric model and stability constants. This behavior could be due to the significant difference in the size of those ions as the effective ionic radius of four-coordinated Zn(II) is about 0.2 Å smaller than those of six-coordinated high-spin Fe(II) and Mn(II).^56^ The smaller ionic radius of the zinc ion could prevent it from interacting firmly with both E_2_ and E_5_, which even though are not far from each other in the peptide sequence, are most probably oriented oppositely to each other, as depicted in the predicted FeoB structure (Figure 2). This could hypothetically be one of the strategies in which the protein displays its specificity in binding appropriate metal ions.^12^

### Comparison of the Thermodynamic Stability of the Complexes

**Figure 9:**
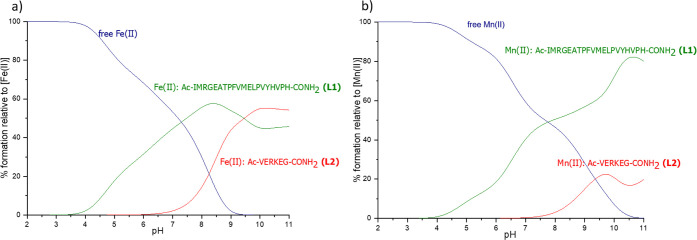
Competition plots of **L1** and **L2** with metal ions. Plot (a) describes Fe(II) and (b) Mn(II) systems, respectively. The concentration of all reagents is 0.5 mM.

Utilizing the stability constants determined by potentiometric titrations, we can discuss the thermodynamic stability of the formed complexes. The stability constants can be used to draw competition plots that reflect speciation in a hypothetical sample containing all reagents in equimolar amounts. In such systems, ligands **L1** and **L2** would compete with each other for the binding of the metal ion (Figure 9).

The competition plots show that for all of the systems, **L1** complexes are more stable than those of **L2** across most of the pH range for Fe(II) and throughout the entire pH range for Mn(II). The slight domination of Fe(II)-**L2** over Fe(II)-**L1** complexes above pH = 9.5 could be due to the possible involvement of amides in metal ion binding at high pH. In **L2**, there are three amides between E_2_ and E_5_ which, if they participate in iron binding, may stabilize the complex. In **L1**, however, there is a proline residue P_19_ close to H_17_ that does not have an amide proton that may be displaced by Fe(II) ions and therefore disturbs the simple stepwise coordination of consecutive amide nitrogens (creating a so-called proline break-point and a macrochelate loop).^57^

**Figure 10:**
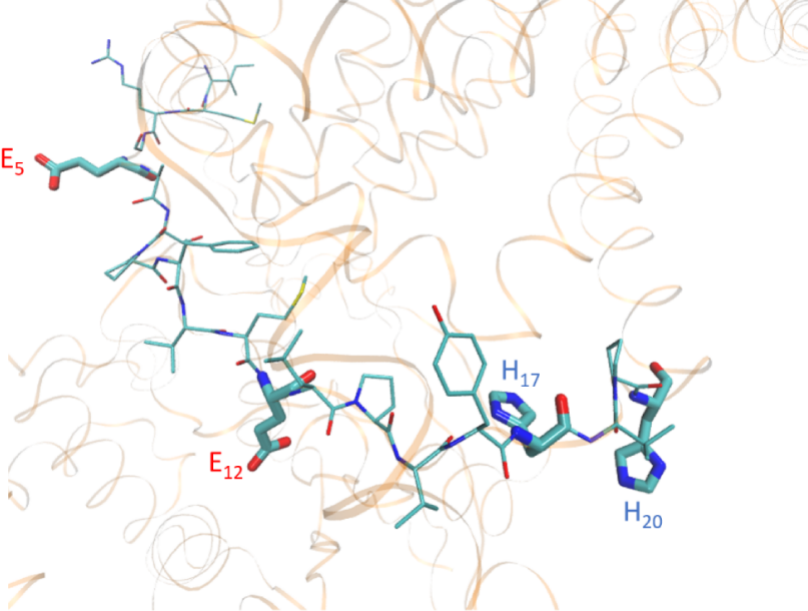
Sequence of **L1** indicated in the structure of*E. coli*FeoB predicted by Alpha Fold. The numbering of the amino acid residues refers to **L1** sequence. Visualized with VMD 1.9.3 software. UniProt ID: P33650.

As **L1** exhibits Fe(II) binding only by histidine residues and **L2** only by glutamic acid residues, this is an interesting example to compare the affinities of divalent iron ions toward nitrogen- and oxygen-based ligands. **L1** complexes start forming at significantly lower pH than **L2** ones, and the metal binding by histidine residues in **L1** seems to be stronger than the metal binding by the glutamic acid residues in **L2**. This is, however, most probably influenced by other factors, such as the folding of the peptide in the solution in a way that prevents the glutamic acid residues from effective binding to the metal ion together with histidine residues if not enough attraction from the metal ion is displayed. It must be noted that even if glutamic acid residues are not directly involved in Fe(II) and Zn(II) binding, they could stabilize the formed complexes, for example, through hydrogen bonds. For **L2**, Fe(II) and Mn(II) bind to E_2_ and E_5_ residues as they are the only ligands available in the sequence of the peptide. This results in very weak complexes. Mn(II), which is a hard acid, prefers hard bases, such as the carboxyl group present in glutamic acid, but also commonly binds to the borderline imidazole groups in proteins.^58^ This is in line with Mn(II) being the only studied metal to directly interact with both glutamic acid and histidine residues in **L1**. Another explanation for the binding to E_5_ and E_12_ in **L1** by Mn(II), but not Fe(II) and Zn(II), could be due to the peptide folding in the solution or the size of the metal ions. As predicted by AlphaFold, the histidine residues, H_17_ and H_20_, are quite distant from the glutamic acid residues, E_5_ and E_12_ (with the numbering referring to the **L1** sequence) (Figure 10). Six-coordinated high-spin Mn(II) displays a bigger value of effective ionic radius (0.82 Å) than six-coordinated high-spin Fe(II) (0.78 Å) and four-coordinated Zn(II) (0.60 Å),^56^ which could help to enable the slightly larger manganese ion to interact with the residues that are further away, for example, with E_5_ which is separated from H_20_ by 14 amino acids. Adapting to different sizes of ionic radii of the metal ions is one of the strategies utilized by proteins to ensure a proper metal ion is bound; however, many other factors may influence the specificity.^12^

**Figure 11:**
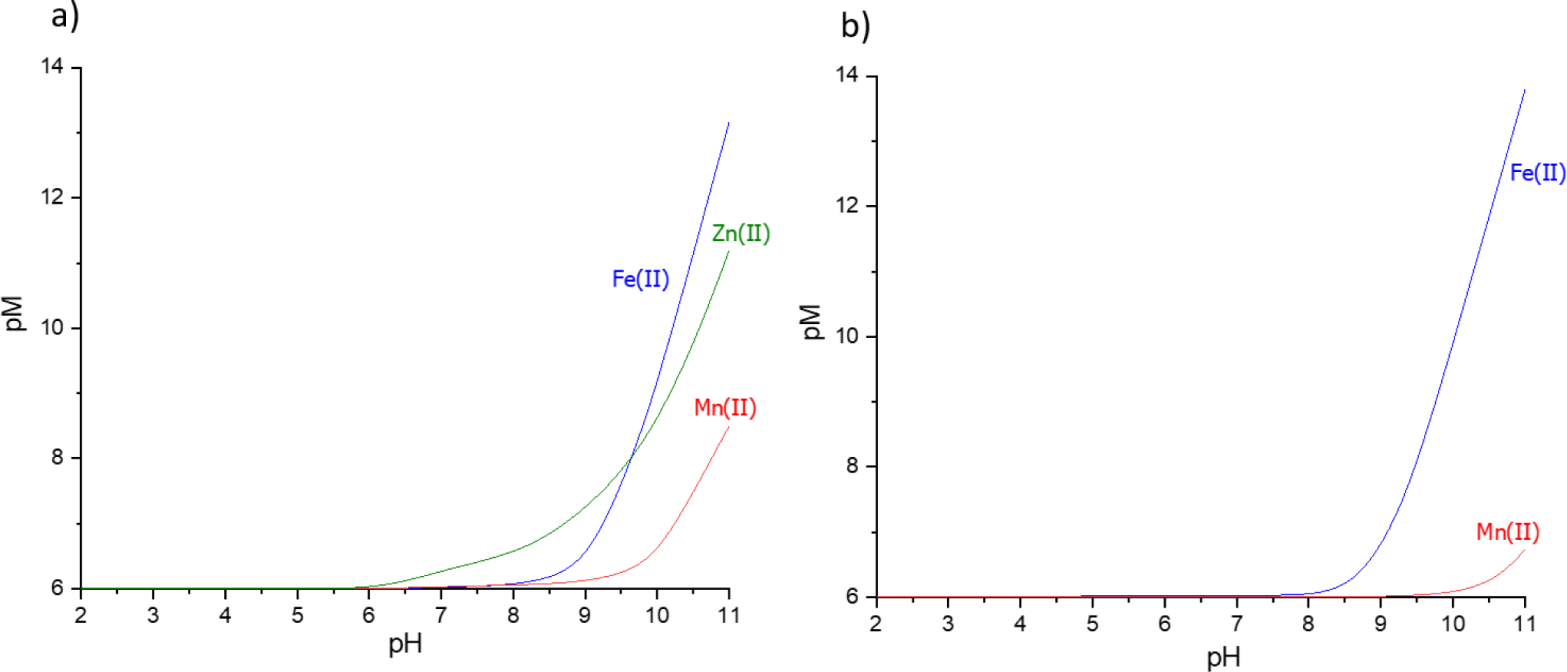
Plots of pM versus pH. Plot (a) represents the **L1** system; plot (b) represents the **L2** system. Conditions: [M]_total_ = 1 × 10^–6^ M, [L]_total_ = 1 × 10^–5^ M.

Another conclusion that can be drawn from the competition plots is that both of the studied ligands form rather weak complexes. The percentage of the free metal ions drops below 50% only above pH = 7.80 for Fe(II) and 7.58 for Mn(II). A relatively small stability of the complexes can also be seen in the plots of pM versus pH (Figure 11). pM, which is a negative logarithm of the free metal ion concentration (−log [M]_free_), is a more biologically relevant factor used for comparing the stability of the complexes.^59,60^ The competition plots are drawn for a hypothetical situation in which all of the reagents are in equimolar amounts, which very rarely happens in the cell. The concentrations used for the pM versus pH plots reflect the cell’s condition better. We have drawn the plots for the conditions: [M]_total_= 1 × 10^–6^ M, which is the concentration of iron in the cell, and a 10-fold excess of the ligand, [L]_total_ = 1 × 10^–5^ M.^61^ The higher the pM value, the lower the concentration of the free metal ion in the solution and thus the greater the stability of the complex and binding ability of the ligand.

Plots shown in Figure 11 highlight the late start of the complexation process for both ligands, consistent with the speciation plots and the competition plots. At pH = 7.4, Zn(II):**L1** system displays a pM value of 6.38 and is the only system at this pH exhibiting a pM value higher than 6, which is the lowest possible value for the [M]_total_ = 1 × 10^–6^ M conditions, meaning 100% of the metal exists in a free form. With the rising pH, the stability of the complexes grows. At pH = 10.0, pM values are 9.23 for Fe(II):**L1** and 9.38 for Fe(II):**L2**. The higher stability of the **L2** complexes of Fe(II) at high pH values is consistent with the competition plots (Figure 9a). A higher stability of the Mn(II):**L1** system than the Mn(II):**L2** system is also consistent with the competition plot (Figure 9b). The stability of the complexes of the metal ions follows the Irving–Williams series of Zn(II)>Fe(II)>Mn(II) for **L1** up to a pH of about 9.5, above which Fe(II) complexes dominate over those of Zn(II). This behavior is observed by us for previously studied systems and can be a result of amide binding in Fe(II) systems, which does not occur in Zn(II) complexes. Fe(II) complexes of **L2** are more stable than those of Mn(II), which is also in line with the Irving–Williams series.

**Figure 12:**
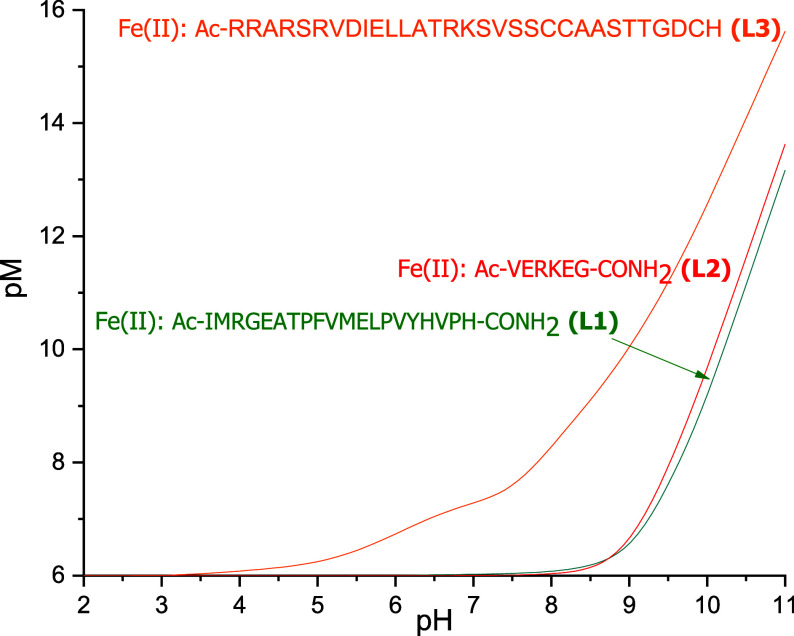
Plot of pFe vs pH for **L1**, **L2**, and **L3**. Conditions: [M]_total_ = 1 × 10^–6^ M, [L]_total_ = 1 × 10^–5^ M.

To have a more comprehensive view of the metal-binding abilities of **L1** (Core CFeoB model) and **L2** (NFeoB ExxE motif model), we decided to compare the stabilities of their complexes with those of the cytoplasmic C-terminal region of the FeoB protein, which we have previously studied.^27^ The ligand named here **L3** contains the whole C-terminal region of the *E. coli* FeoB, with the sequence: Ac_743_-RRARSRVDIELLATRKSVSSCCAASTTGDCH_773_. Figure 12 shows a pFe versus pH plot between **L1**, **L2**, and **L3**.

The competition plot shown in Figure 12 shows a clear dominance of **L3** throughout the pH range over ligands **L1** and **L2** studied in this work. At pH = 7.4, the pFe value for **L3** is 7.51, while for **L1** and **L2,** the values are only slightly above 6, meaning that for these two ligands, Fe(II) exists almost entirely in the form of free ions at this pH. There are three cysteine residues, one histidine residue, two aspartic acid residues, and one glutamic acid residue in the sequence of **L3**. The significantly greater stability of its complexes can most probably be explained by the presence of the cysteine residues, which, as soft bases, are good ligands for borderline acid Fe(II). Five arginine residues present in **L3** can also stabilize the complexes by hydrogen bonds. The plots of pMn and pZn were very similar to those of Fe(II) and showed significantly greater stability of the **L3** complexes.

To discuss the thermodynamic stability of **L1** and **L2** complexes in even more depth, we decided to calculate their dissociation constants, which can be defined as , and refer to the overall equilibrium: ML ⇄ M + L.^62^ The value of the dissociation constant is frequently used to compare the stability of the metal complexes of proteins. The lower the value of *K*_*d*_, the stronger the ligand binds the metal ion. We have decided to compare the values of dissociation constants for the complexes of **L1**, **L2**, and **L3** studied in this and in previous work^27^ with values available in the literature for various divalent metal ions transporting bacterial proteins, e.g., MtsA and YfeA Fe(II) transporters,^63,64^ Znu and TroA Zn(II) transporters,^65,66^ YfeA and MntH Mn(II) transporters,^64,67^ as well as with some regulatory proteins, such as Fur (Ferric Uptake Regulator)^68^ and MntR,^69^ which is a regulatory protein for manganese homeostasis. The dissociation constant values are collected in Table 5.

**Table 5:** Comparison of *K*_d_ Values for Studied and Biological Ligands for Fe(II), Zn(II), and Mn(II)^a^

Ligand	Fe(II)	Mn(II)	Zn(II)	ref.
**L1** (*E. coli* Core CFeoB)	1.88 × 10^–4^	1.35 × 10^–4^	1.07 × 10^–5^	This work
**L2** (*E. coli* ExxE motif)	2.21 × 10^–3^	1.31 × 10^–2^	-	This work
**L3***(E. coli* C-terminal FeoB)	4.75 × 10^–7^	7.02 × 10^–7^	6.31 × 10^–8^	(27)
*E. coli* Fur	1.2 × 10^–6^	2.4 × 10^–5^	1.4 × 10^–10^	(68)
*S. pyogenes* MtsA	4.3 × 10^–6^	-	-	(63)
*B. subtilis* MntR	-	0.2 × 10^–6^–2 × 10^–6^	-	(69)
*Y. pestis* YfeA	-	1.78 × 10^–8^	6.6 × 10^–9^	(64)
*T. pallidum* TroA	-	7.1 × 10^–9^	2.25 × 10^–8^	(66)
*D. radiodurans* MntH	-	1.9 × 10^–4^	-	(67)
*Synechocystis* ZnuA	-	-	7.3 × 10^–9^	(65)

a *K*_*d*_ values calculated as  at pH = 7.0. [M] refers to the concentration of the free metal ion present at this pH; [L] refers to the sum of the concentrations of all ligand species present at this pH; [ML] refers to the sum of the concentrations of all metal-ligand complex species present at this pH.

The dissociation constant values for the studied ligands **L1** and **L2** are significantly higher than those of the previously studied fragment **L3** of *E. coli* C-terminal FeoB, meaning that the stability of their complexes is much lower. Compared with data available in the literature for other Fe(II), Mn(II), and Zn(II)-binding proteins, affinities of **L1** and **L2** toward these metal ions are also significantly lower. Only the value of the dissociation constant for Mn(II):**L1** system (*K*_*d*_ = 1.35 × 10^–4^) is comparable with the MntH:Mn(II) system (*K*_*d*_ = 1.9 × 10^–4^). However, this is also due to the quite low dissociation constant reported for this system, with respect to other values reported for Mn(II) complexes with proteins. It is worth noting that at pH = 7.0, **L1** has a slightly higher affinity toward Mn(II) than Fe(II), characterized by a lower *K*_*d*_ value (1.35 × 10^–4^ and 1.88 × 10^–4^, respectively). The difference between the values is small but suggests that the **L1** fragment of the Core CFeoB region could bind both Fe(II) and Mn(II) with similar strength. Relatively high *K*_*d*_ values determined for **L1** and **L2** systems suggest weak affinity of the Core CFeoB and ExxE motif toward the studied metal ions; however, the stability of the complex is not always the deciding factor for metal complexation in proteins. Metal ion transporters usually display lower affinity toward the metal than, for example, metal chelators, and other factors such as conformational changes of the protein likely play an important role in accommodating the binding site toward the right metal ion, ensuring proper metalation.^12^ Furthermore, one must bear in mind that the number of interactions present in the protein, which could stabilize the metal complex, is vastly greater than in our peptidic model studies.

## Conclusions

**Figure 13:**
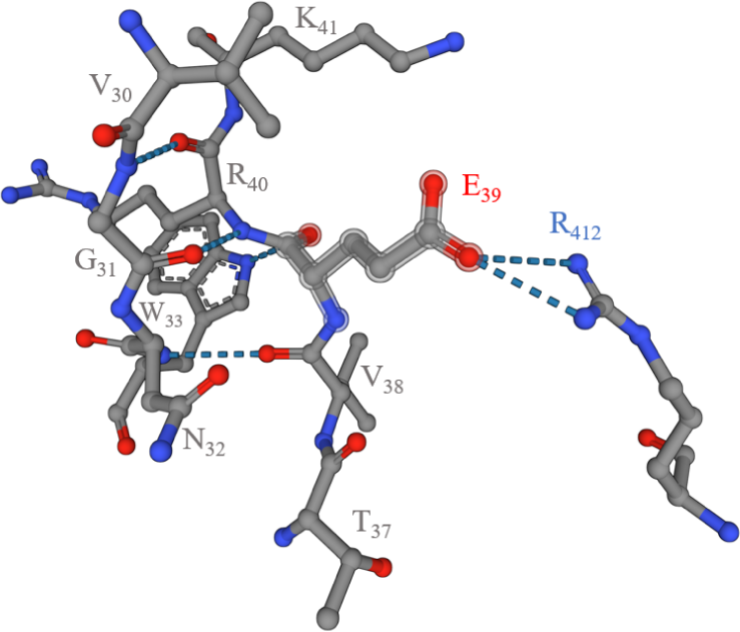
Surroundings of the E_39_ residue in the structure of *E. coli* FeoB (UniProt ID: P33650) predicted by an AlphaFold. The residues shown are no further than 5 Å from E_39_.

Both of the studied regions of the *E. coli* FeoB protein, the Core CFeoB region and the ExxE motif located in the cytoplasmic NFeoB are proposed in the literature as putative Fe(II) binding sites. However, all of the data discussed in the previous sections indicate that both **L1** and **L2** form rather weak complexes with the studied metal ions, probably due to the insufficient number of possible metal-binding residues to ensure strong complexation. It must be noted that **L1** and **L2** act as models for the Core CFeoB region and ExxE motif, respectively, and their capability of metal-binding *in vivo* cannot be fully elucidated through this type of solution studies. As potentiometric titrations can be used only for peptides consisting of a limited number of amino acids, the whole Core CFeoB region and NFeoB domain cannot be studied in such a way. As of now, no crystal structure of the whole FeoB protein has been obtained; however, tools such as AlphaFold can simulate the protein folding and structure, providing information about the vicinity of the selected peptide fragments in the structure of the whole protein.^36,37^ As the protein folds, amino acids that are far from each other in the protein sequence can interact and stabilize the forming metal complexes, enhancing their stability. For example, E_2_ of the **L2** ligand (E_39_ in the protein sequence) is predicted to form a hydrogen bond with an arginine residue, R_412_ (Figure 13), which is close in the sequence of the protein with aspartic acid D_415_ and glutamic acid E_419_. Regarding **L1**, H_17_ (H_493_ in the protein sequence) is spatially close to E_368_ and D_369_, which could participate in metal complexation. It must be noted that the structure of the protein provided by AlphaFold is just a prediction; nevertheless, the additional residues in the protein structure could improve the metal-binding properties of the residues, making the Core CFeoB region and the ExxE motif more effective binding sites for the studied metals than the results for the individual peptides in this study would suggest. The idea of additional residues interacting with the ExxE motif and facilitating proper Fe(II) binding has also been discussed by Hung et al.^35^

However, this does not necessarily mean that the metal complexation abilities of the studied regions in the native protein are significantly higher than those expressed by the ligands **L1** and **L2** in this study. If the stability of the complexes with the metal ions is low, it could be due to the biological role of these regions. As *E. coli* FeoB is a large transmembrane protein, the process of Fe(II) transport through the inner membrane is most probably carried out in a multistep fashion, with multiple regions of the protein taking part in transferring the metal ion from the periplasm to the cytoplasm. If that is true, some of these regions should bind Fe(II) in a labile way, in the form of weak complexes ensuring a rapid process of binding the metal ion and its dissociation, facilitating a smooth translocation of Fe(II). However, to see if that is the case for the Core CFeoB region and the ExxE motif, further studies are required. Nevertheless, solution studies such as those described in this work can shed light on the coordination chemistry of the FeoB fragments and the coordination chemistry of the peptide complexes of the studied metal ions, particularly for Fe(II) and Mn(II), for which this type of research is still lacking in the literature.

Further fragments of *E. coli* FeoB, speculated to possess a metal-binding function, such as Gate 1 and Gate 2 regions, are currently being studied in our laboratories to present a comprehensive view of the coordination chemistry of the most plausible Fe(II)-binding regions of the FeoB protein.

## Supporting Information Available

Mass spectra of the studied systems, additional NMR spectra, free ligand speciation plots, and tables containing metal hydrolysis constants and the major signals observed in the mass spectra (PDF)
