# Supplementary material for: The Coordination Chemistry of Two Peptidic Models of NFeoB and Core CFeoB Regions of FeoB Protein: Complexes of Fe(II), Mn(II), and Zn(II)
Source: Inorg Chem. 2025 Mar 6;64(10):5038–52. doi: 10.1021/acs.inorgchem.4c05111 (PMC11920956; doi:10.1021/acs.inorgchem.4c05111)
Supplement: Supplementary file 1 — ic4c05111_si_001.pdf [file ic4c05111_si_001.pdf]

Supporting information for:

The coordination chemistry of two peptidic models of NFeoB and Core CFeoB regions of FeoB protein: complexes of Fe(II), Mn(II), and Zn(II)

Bartosz Orzel[a], Malgorzata Ostrowska[a], Slawomir Potocki[a], Maria Antonietta Zoroddu[b], Henryk Kozlowski[a,c], Massimiliano Peana[b], and Elzbieta Gumienna-Kontecka[a]*

[a] Faculty of Chemistry, University of Wrocław, 50-383 Wrocław, Poland

[b] Department of Chemical, Physical, Mathematical and Natural Sciences, University of Sassari, 07100 Sassari, Italy

[c] Faculty of Health Sciences, University of Opole, Katowicka 68, 45-060 Opole, Poland

*Email: elzbieta.gumienna-kontecka@uwr.edu.pl

Table S1. Hydrolysis constants for Mn(II), Zn(II) and Fe(II) ions for I = 0.1M ionic strength, T= 298 K.[a]

Species	log β
**Mn(II)**	
**$Mn(OH)^{+}$**	-10.78
**$Mn(OH)_2$**	-22.39
**$Mn(OH)_3^{-}$**	-34.34
**$Mn(OH)_4^{2-}$**	-47.82
**Zn(II)**	
**$Zn(OH)^{+}$**	-9.12
**$Zn(OH)_2$**	-18.08
**$Zn(OH)_3^{-}$**	-27.97
**$Zn(OH)_4^{2-}$**	-39.50
**Fe(II)**	
**$Fe(OH)^{+}$**	-9.63
**$Fe(OH)_2$**	-20.73
**$Fe(OH)_3^{-}$**	-32.68

[a] The hydrolysis constants for zero ionic strength[1] were recalculated to 0.1 M ionic strength with an appropriate formula.[2]

(1) Baes, C.F.; Mesmer, R.S. The Hydrolysis of Cations. John Wiley & Sons, 1976.
(2) Brown, P. L.; Ekberg, C. *Hydrolysis of Metal Ions*; John Wiley & Sons, 2016.

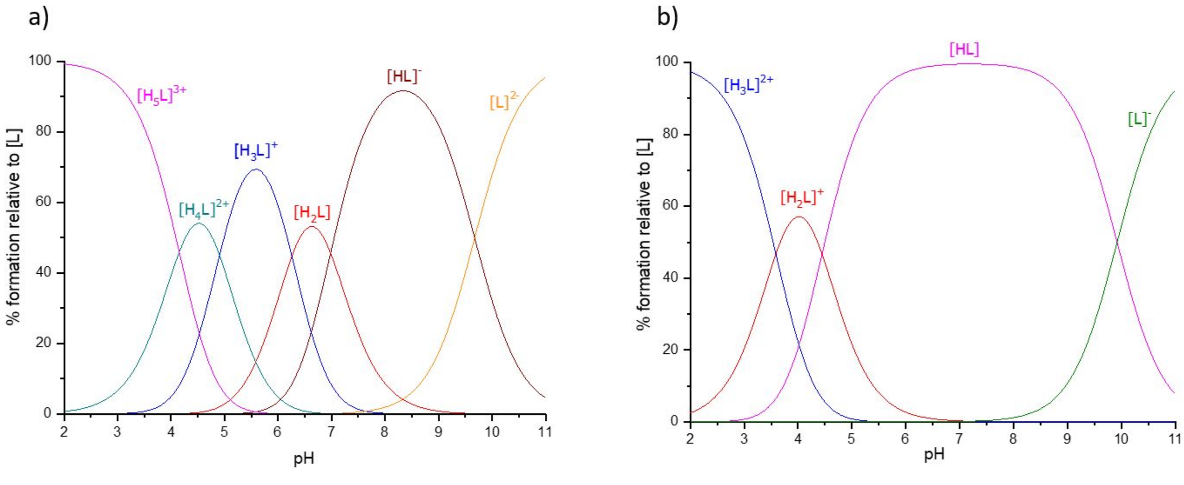

Figure S1. Distribution diagrams of the proton complexes of the ligands: a) **L1**; b) **L2**.

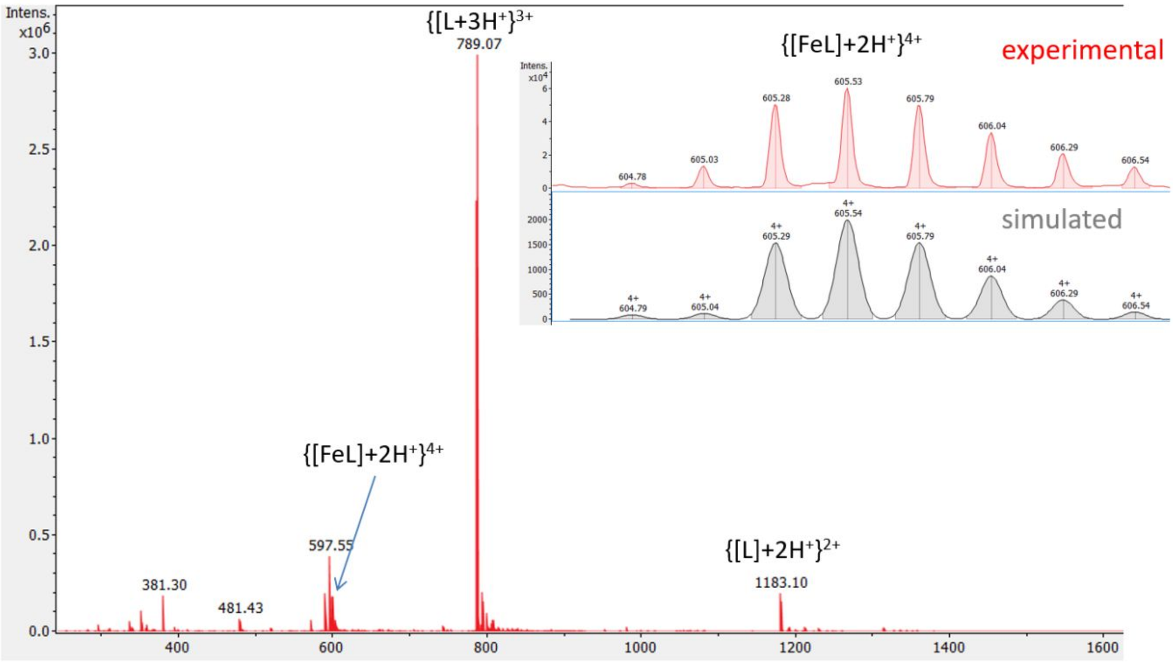

Figure S2. ESI-MS spectrum of the Fe(II):**L1** system. Fe(II):L = 1:1. The simulated and experimental isotopic distribution of the signal at m/z= 604.78 is shown on the right.

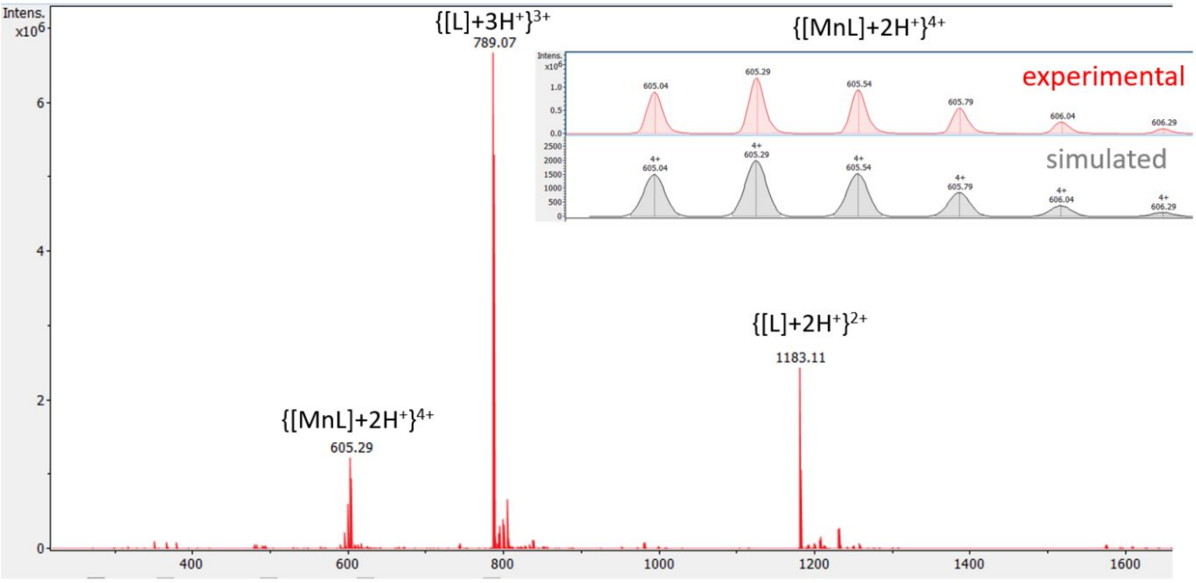

Figure S3. ESI-MS spectrum of the Mn(II):**L1** system. Mn(II):L = 1:1. The simulated and experimental isotopic distribution of the signal at m/z= 605.04 is shown on the right.

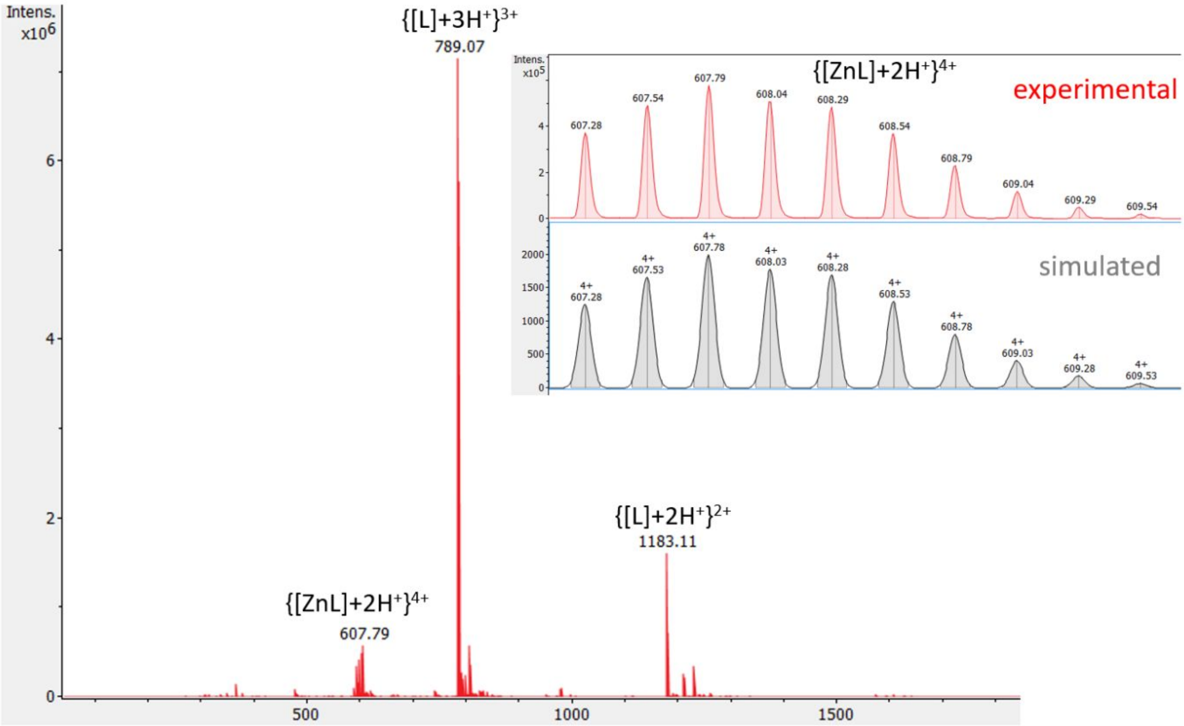

Figure S4. ESI-MS spectrum of the Zn(II):**L1** system. Zn(II):L = 1:1. The simulated and experimental isotopic distribution of the signal at m/z= 607.28 is shown on the right.

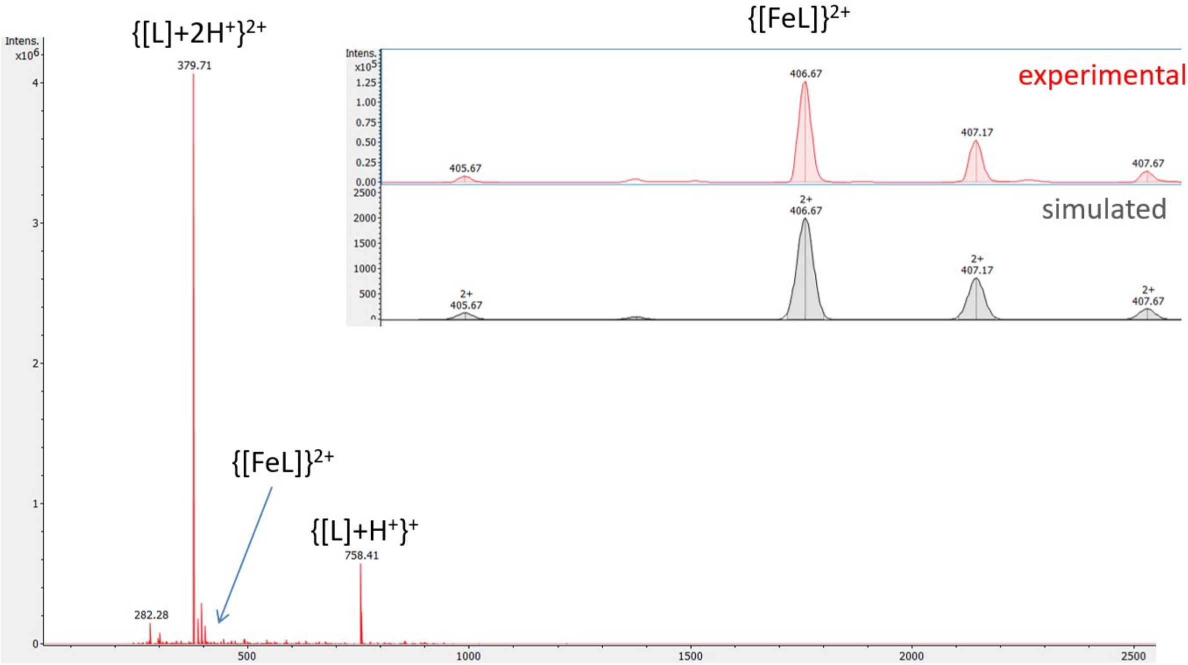

Figure S5. ESI-MS spectrum of the Fe(II):**L2** system. Fe(II):L = 1:1. The simulated and experimental isotopic distribution of the signal at m/z= 405.67 is shown on the right.

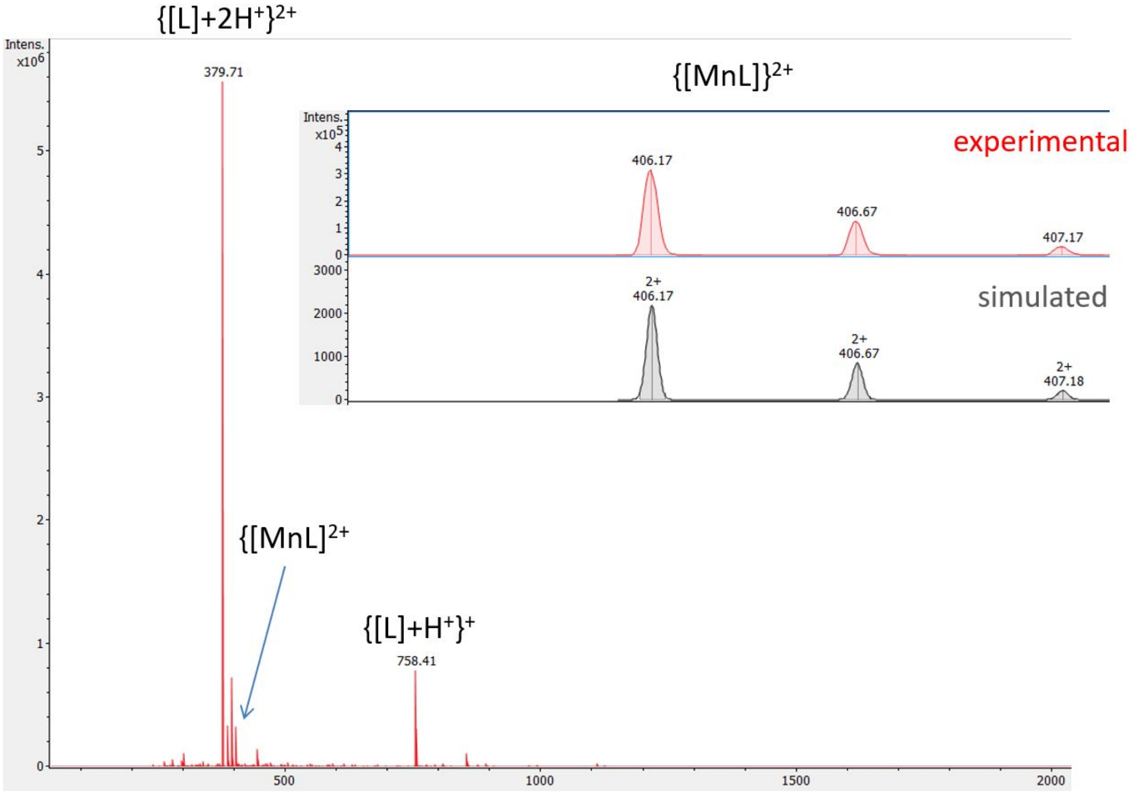

Figure S6. ESI-MS spectrum of the Mn(II):**L2** system. Mn(II):L = 1:1. The simulated and experimental isotopic distribution of the signal at m/z= 406.17 is shown on the right.

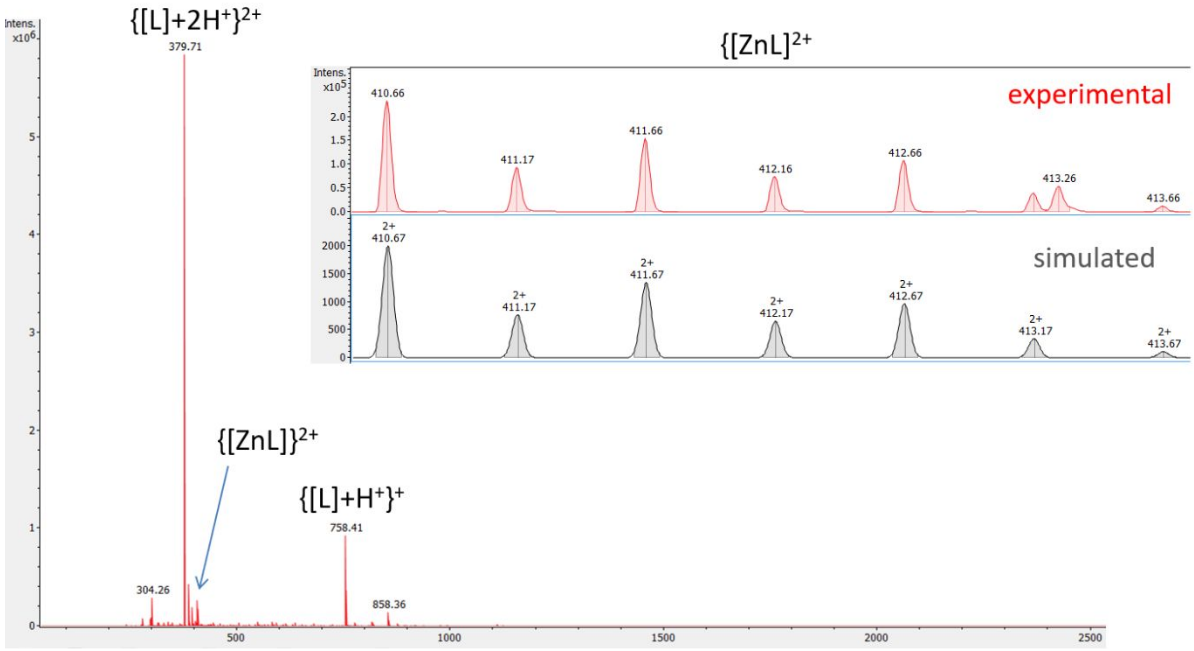

Figure S7. ESI-MS spectrum of the Zn(II):**L2** system. Mn(II):L = 1:1. The simulated and experimental isotopic distribution of the signal at m/z= 410.66 is shown on the right.

Table S2. Intensity maxima of the major complexes and adduct ions observed by ESI-MS for each M(II):peptide system, M:L=1:1.

System	Ion	m/*z* experimental	m/*z* simulated
Fe(II):**L1**	$\{[FeL]+2H^{+}\}^{4+}$	604.78	604.79
	$\{[L]+3H\}^{3+}$	788.74	788.74
	$\{[L]+2H^{+}\}^{2+}$	1182.60	1182.60
Mn(II):**L1**	$\{[MnL]+2H^{+}\}^{4+}$	605.04	605.04
	$\{[L]+3H^{+}\}^{3+}$	788.74	788.74
	$\{[L]+2H^{+}\}^{2+}$	1182.60	1182.60
Zn(II):**L1**	$\{[ZnL]+2H^{+}\}^{4+}$	607.28	607.28
	$\{[L]+3H^{+}\}^{3+}$	788.74	788.74
	$\{[L]+2H^{+}\}^{2+}$	1182.60	1182.60
Fe(II):**L2**	$\{[FeL]\}^{2+}$	405.67	405.67
	$\{[L]+2H^{+}\}^{2+}$	379.71	379.71
	$\{[L]+H^{+}\}^{+}$	758.41	758.42
Mn(II):**L2**	$\{[MnL]\}^{2+}$	406.17	406.17
	$\{[L]+2H^{+}\}^{2+}$	379.71	379.71
	$\{[L]+H^{+}\}^{+}$	758.41	758.42
Zn(II):**L2**	$\{[ZnL]\}^{2+}$	410.66	410.67
	$\{[L]+2H^{+}\}^{2+}$	379.71	379.71
	$\{[L]+H^{+}\}^{+}$	758.41	758.42

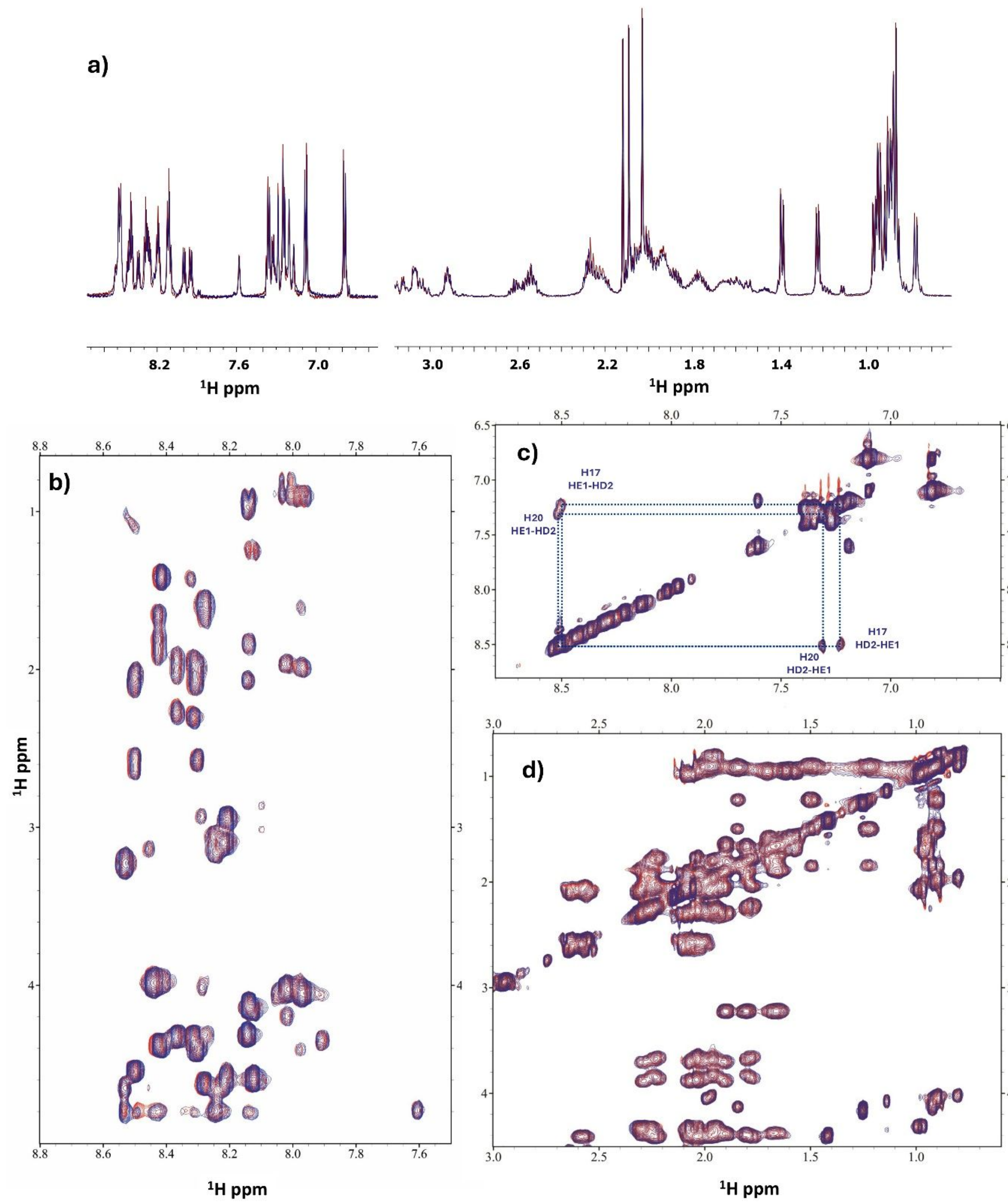

Figure S8. a) Comparison of ^{1}H spectra and (b, c and d) selection of ^{1}H−^{1}H TOCSY spectra for the free peptide **L1** (red) and Fe(II):**L1** system (blue) at 1:3 molar ratio and pH = 5.5. Perturbed signals are indicated in red.

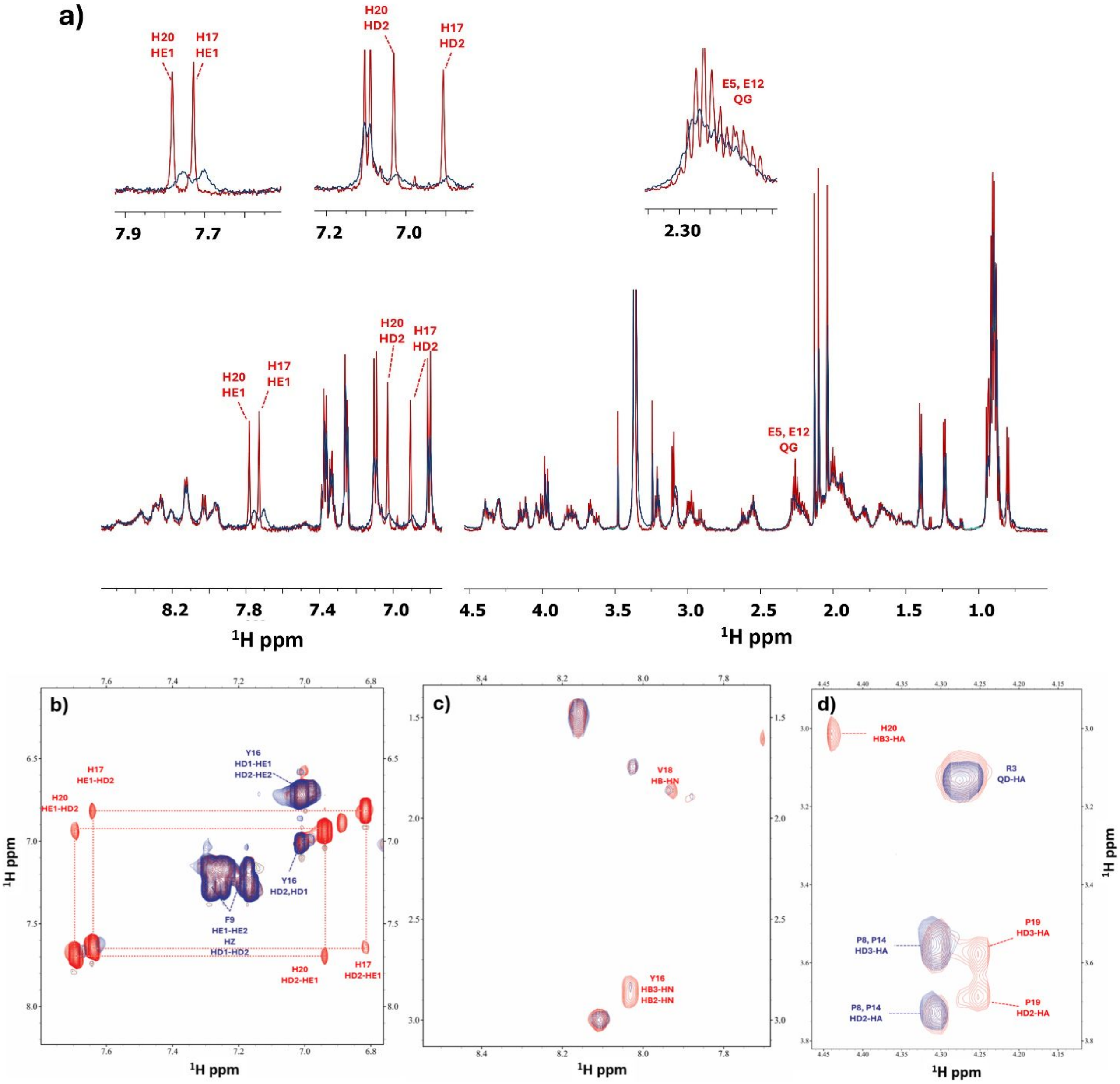

Figure S9. a) Comparison of ^{1}H spectra and (b, c and d) selection of ^{1}H−^{1}H TOCSY spectra for the free peptide **L1** (red) and Fe(II):**L1** system (blue) at 1:3 molar ratio and pH = 7.7. Perturbed signals are indicated in red.

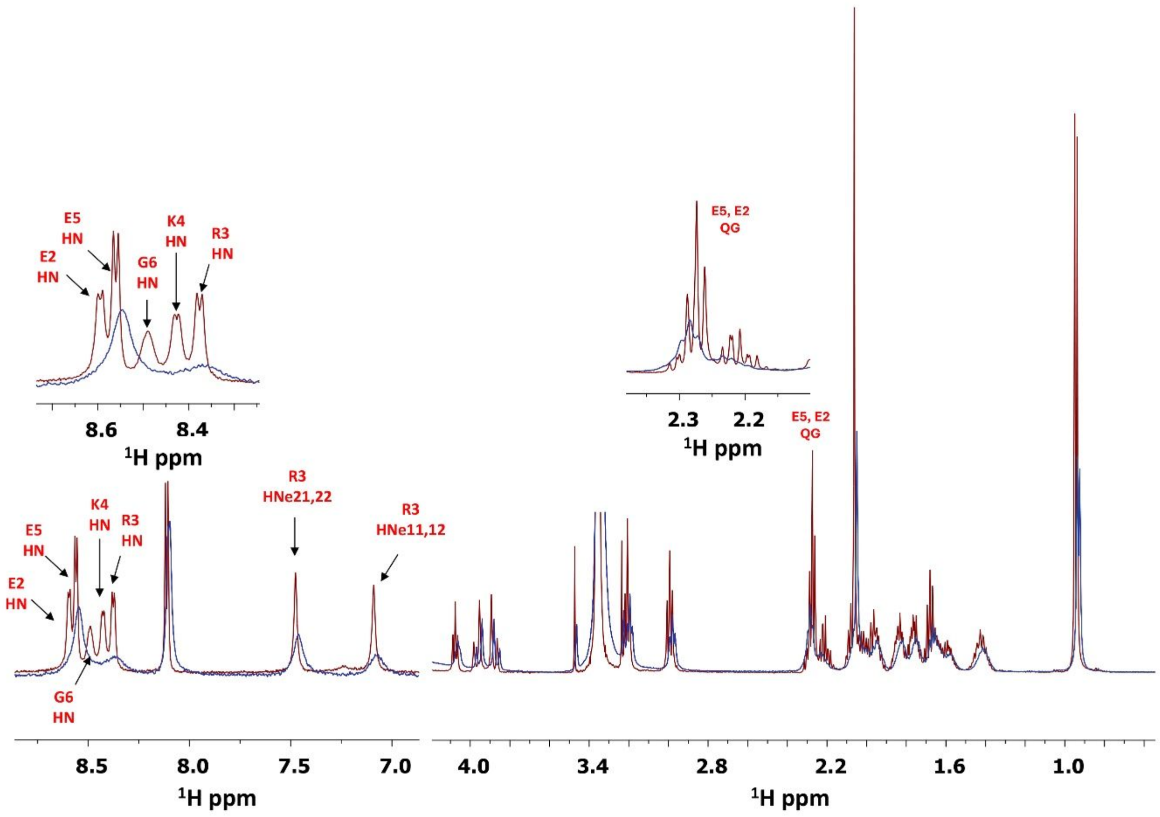

Figure S10. Comparison of ^{1}H spectra and for the free peptide **L2** (red) and Fe(II):**L2** system (blue) at 1:3 molar ratio and pH = 8.2. Perturbed signals are indicated in red.

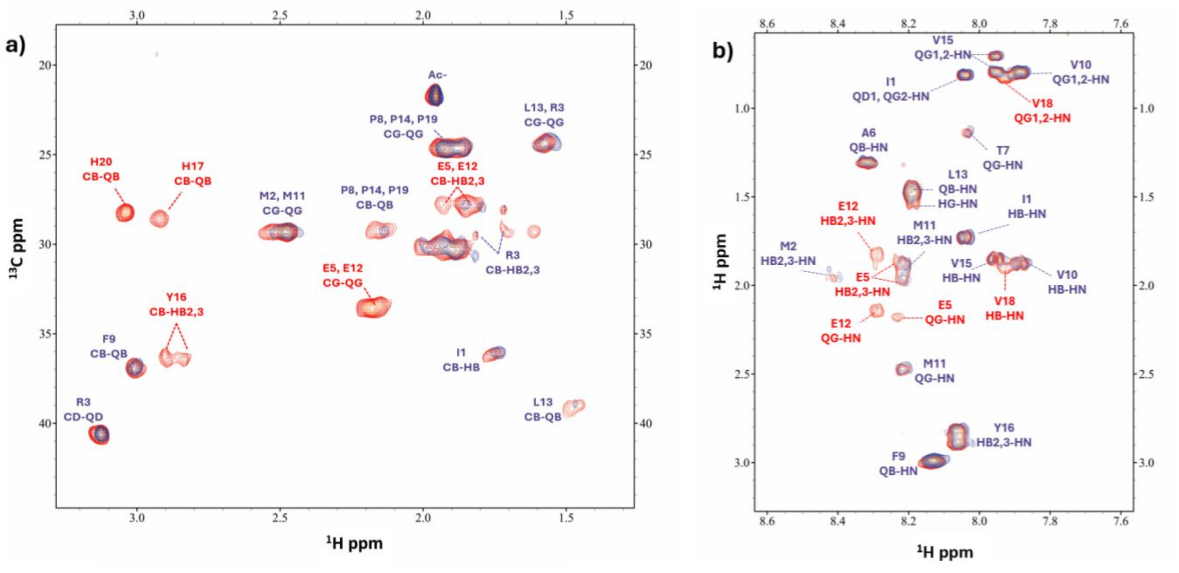

Figure S11. a) Comparison of a selected aliphatic region in the $^{1}H-^{13}C$ HSQC spectra and (b) a selected aromatic region in the $^{1}H-^{1}H$ TOCSY spectra for the free peptide **L1** (red) and Mn(II):**L1** system (blue) at 1:50 molar ratio and pH = 7.0. Perturbed signals are indicated in red.

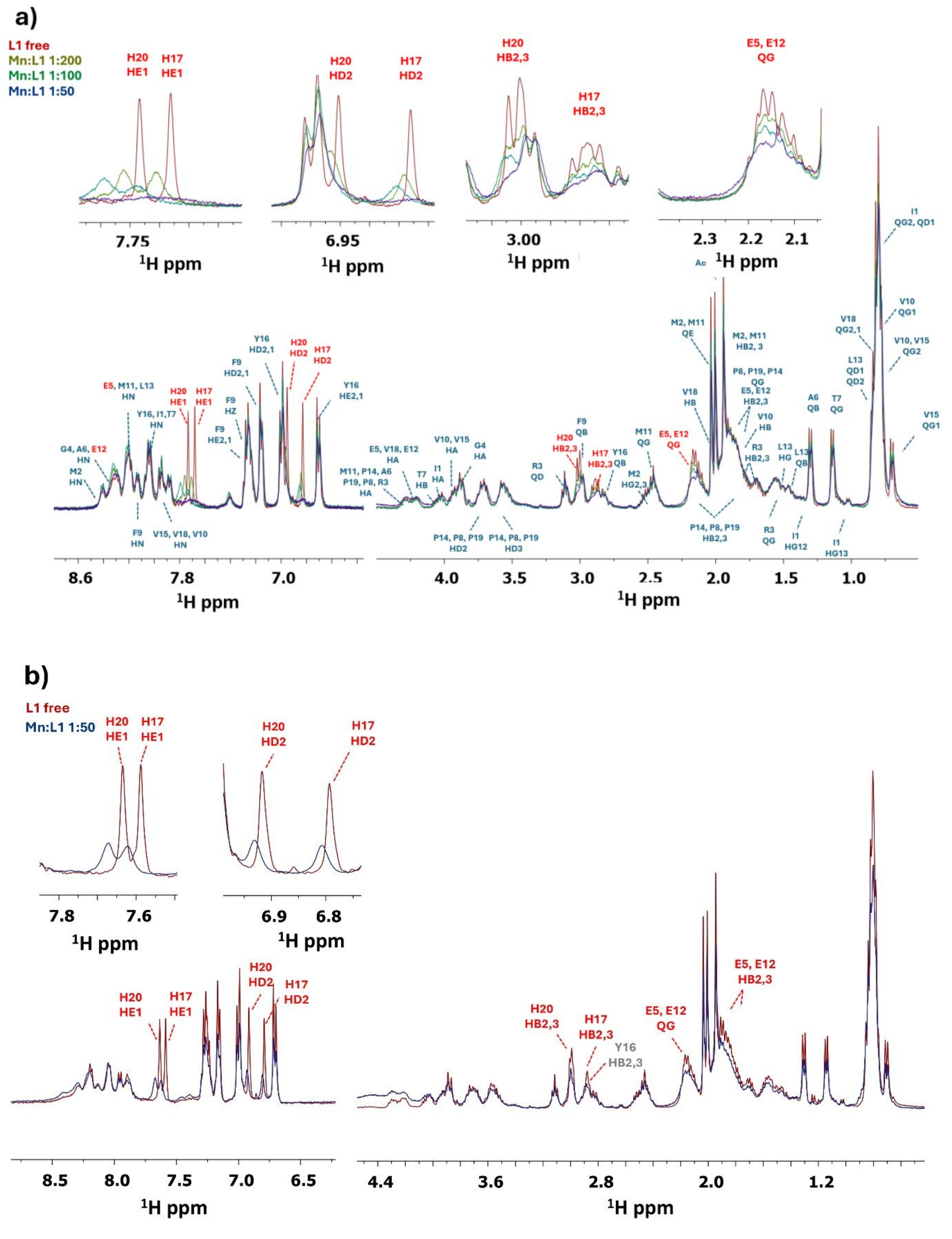

Figure S12. Comparison of ^{1}H spectra for the free peptide **L1** (red) with those following the sequential addition of Mn(II) at a) pH = 7.0 and b) pH = 7.8. Perturbed signals are indicated in red.

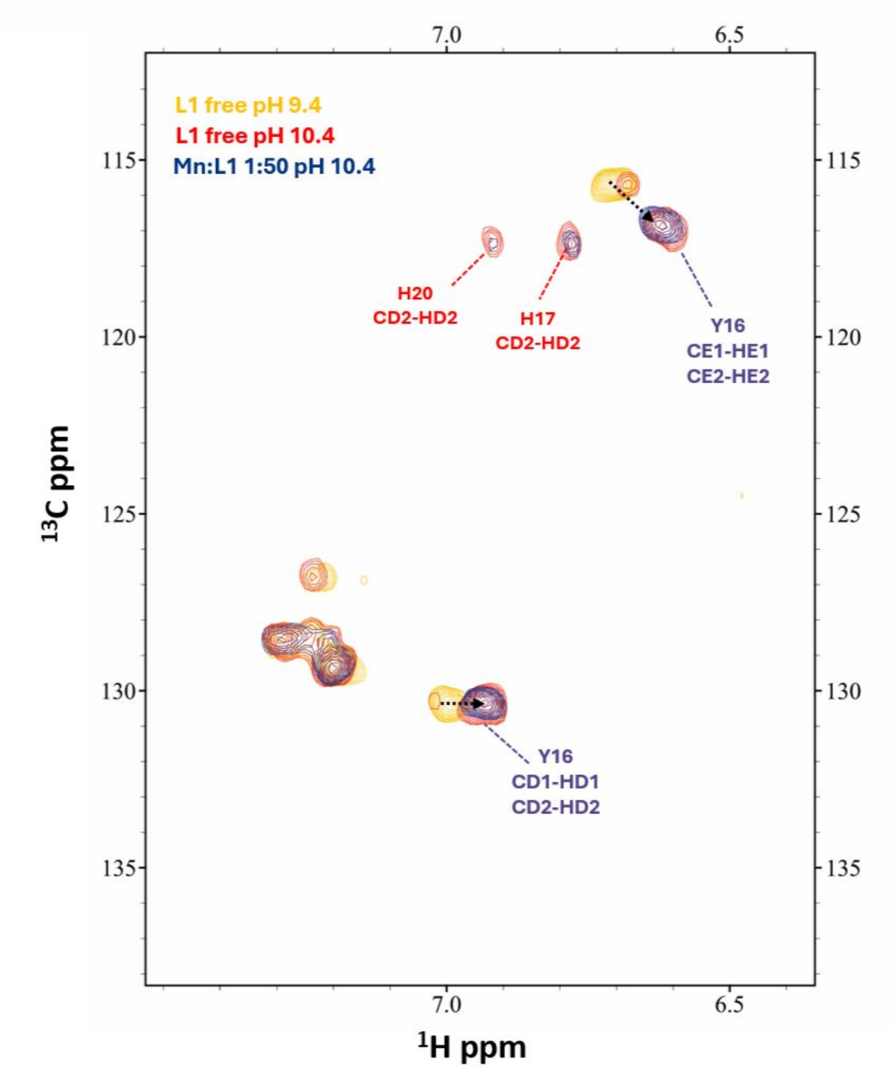

Figure S13. Comparison of an aromatic region in the ^{1}H–^{13}C HSQC spectra for the free peptide **L1** at pH 9.4 (yellow) and pH 10.4 (red) and Mn(II):**L1** system (blue) at 1:50 molar ratio, pH = 10.4, in SDS. The arrow indicates the shift of the Tyr aromatic proton due to -OH deprotonation, and the lack of coordination is confirmed by the signals that are still present in the Mn(II):L1 system.

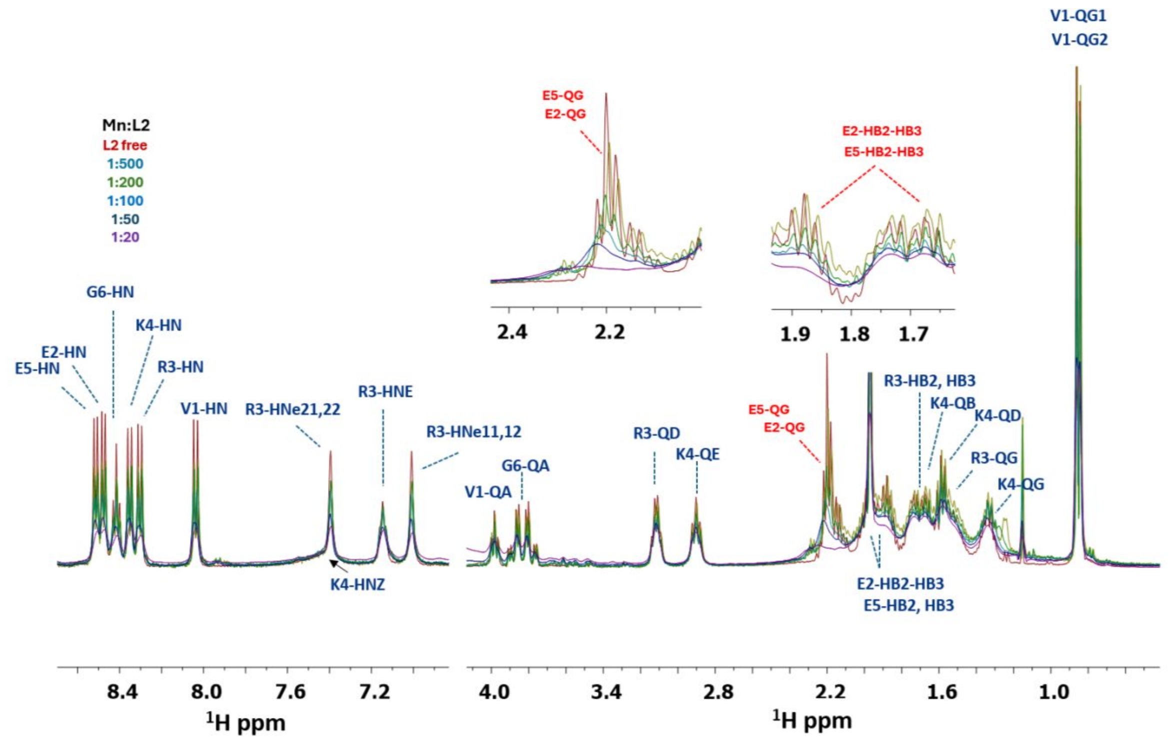

Figure S14. Comparison of ^{1}H spectra for the free peptide **L2** (red) with those following the sequential addition of Mn(II) at pH=5.1. Perturbed signals are indicated in red.

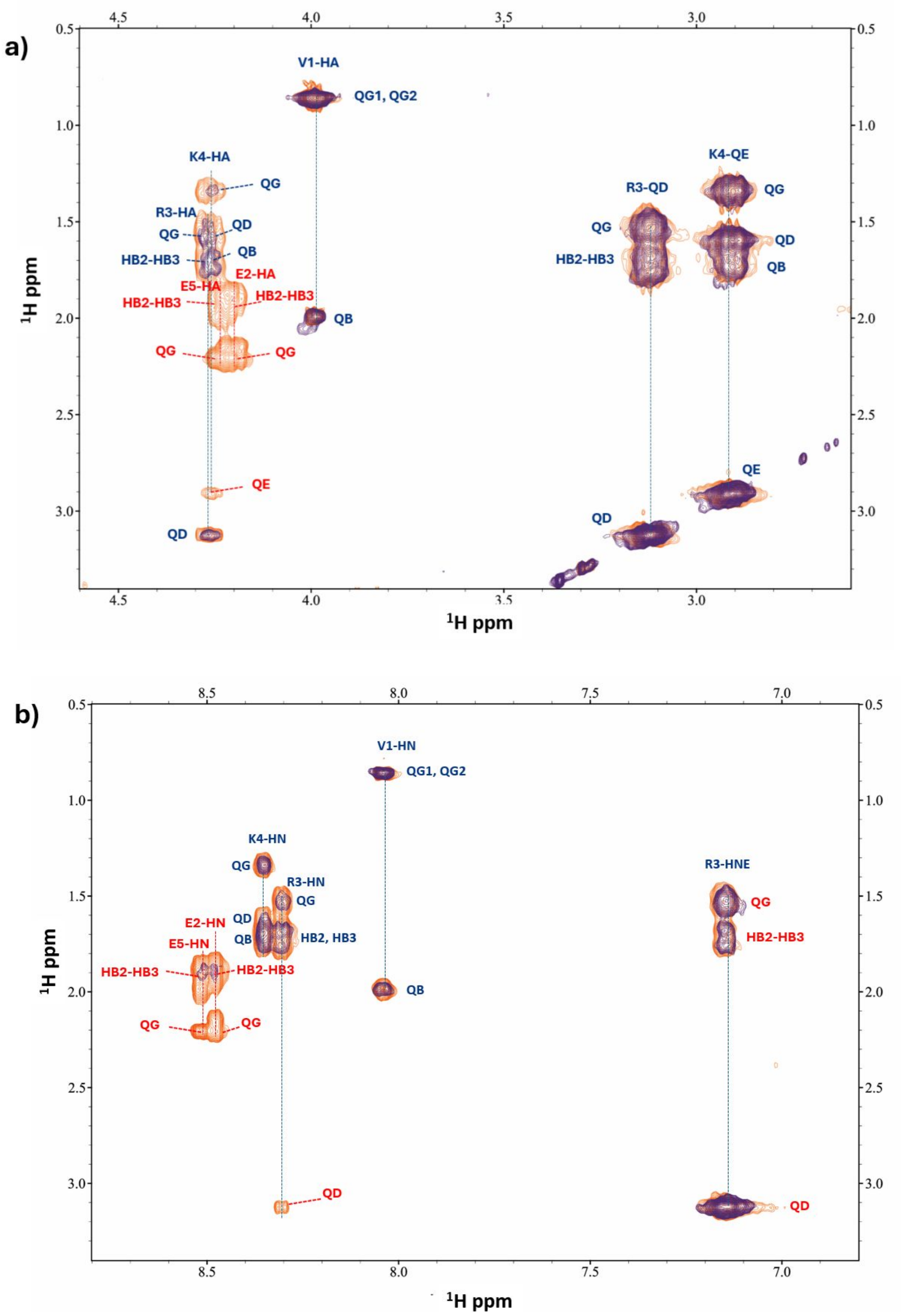

Figure S15. a) Comparison of a selected aliphatic a) and aromatic b) region in the $^{1}H-^{1}H$ TOCSY spectra for the free peptide **L2** (red) and Mn(II):**L2** system (blue) at 1:50 molar ratio and pH = 5.1. Perturbed signals are indicated in red.

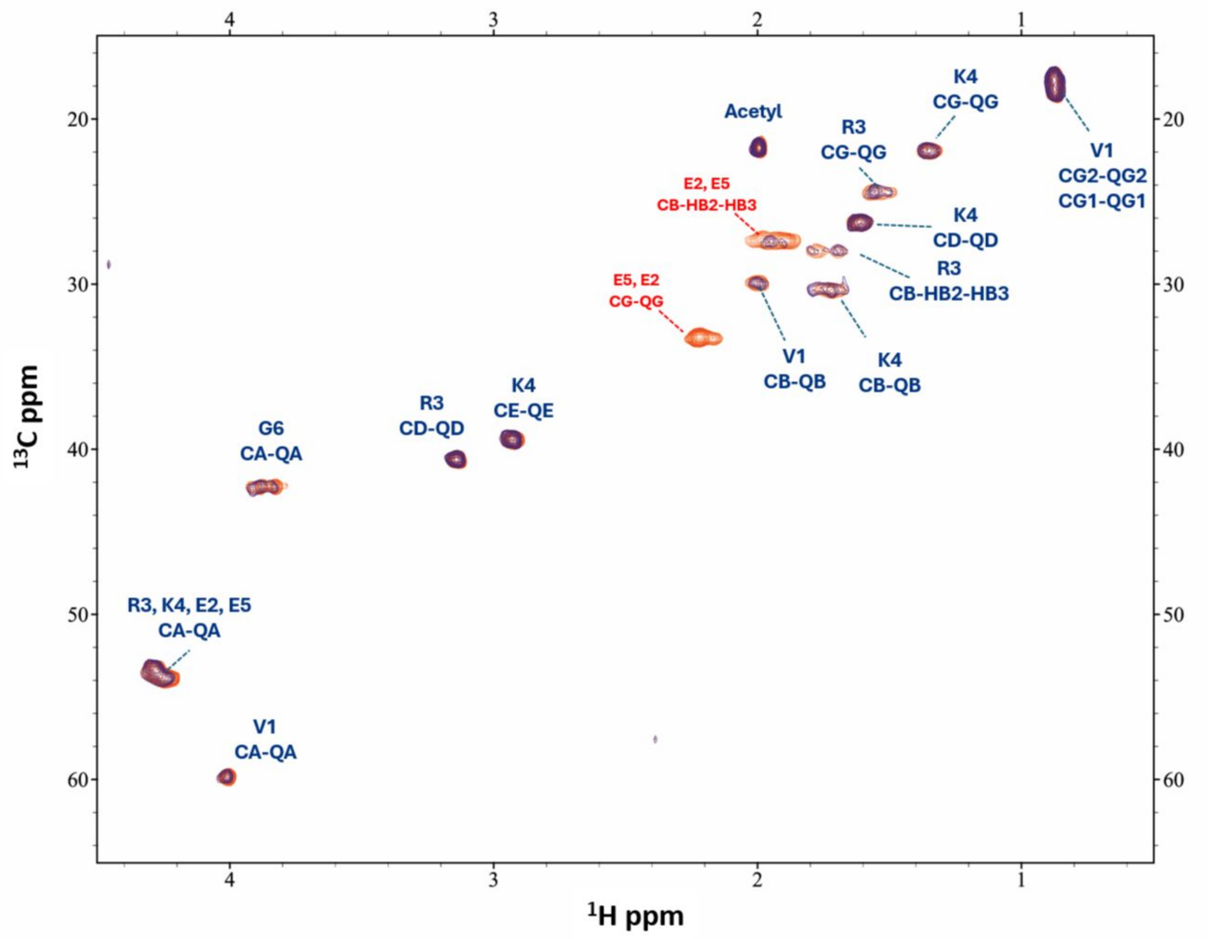

Figure S16. a) Comparison of the aliphatic region in the ^{1}H–^{13}C HSQC spectra and (b) a selected aromatic region in the ^{1}H–^{1}H TOCSY spectra for the free peptide **L2** (red) and Mn(II):**L2** system (blue) at 1:50 molar ratio and pH = 7.0. Perturbed signals are indicated in red.

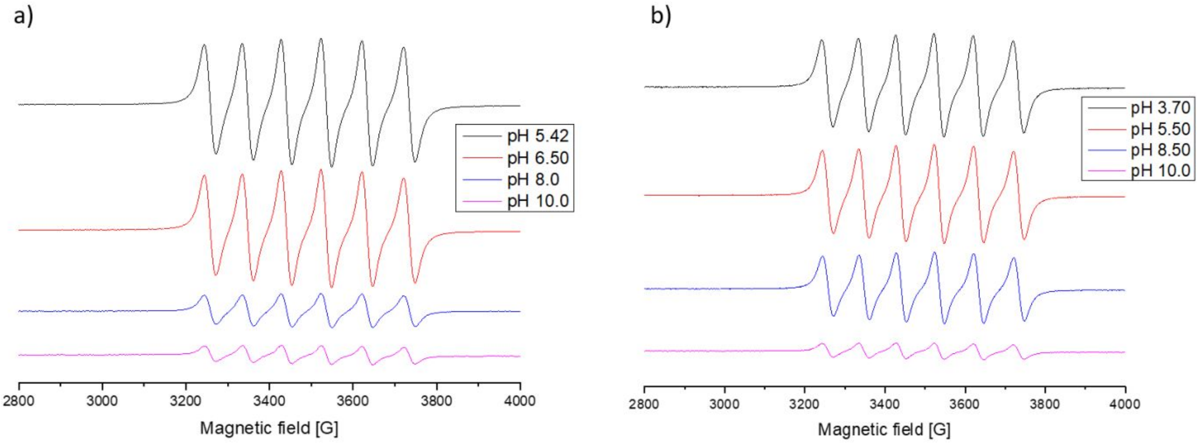

Figure S17. EPR spectra recorded at room temperature at various pH values for: a) Mn(II):**L1** system; b) Mn(II):**L2** system.

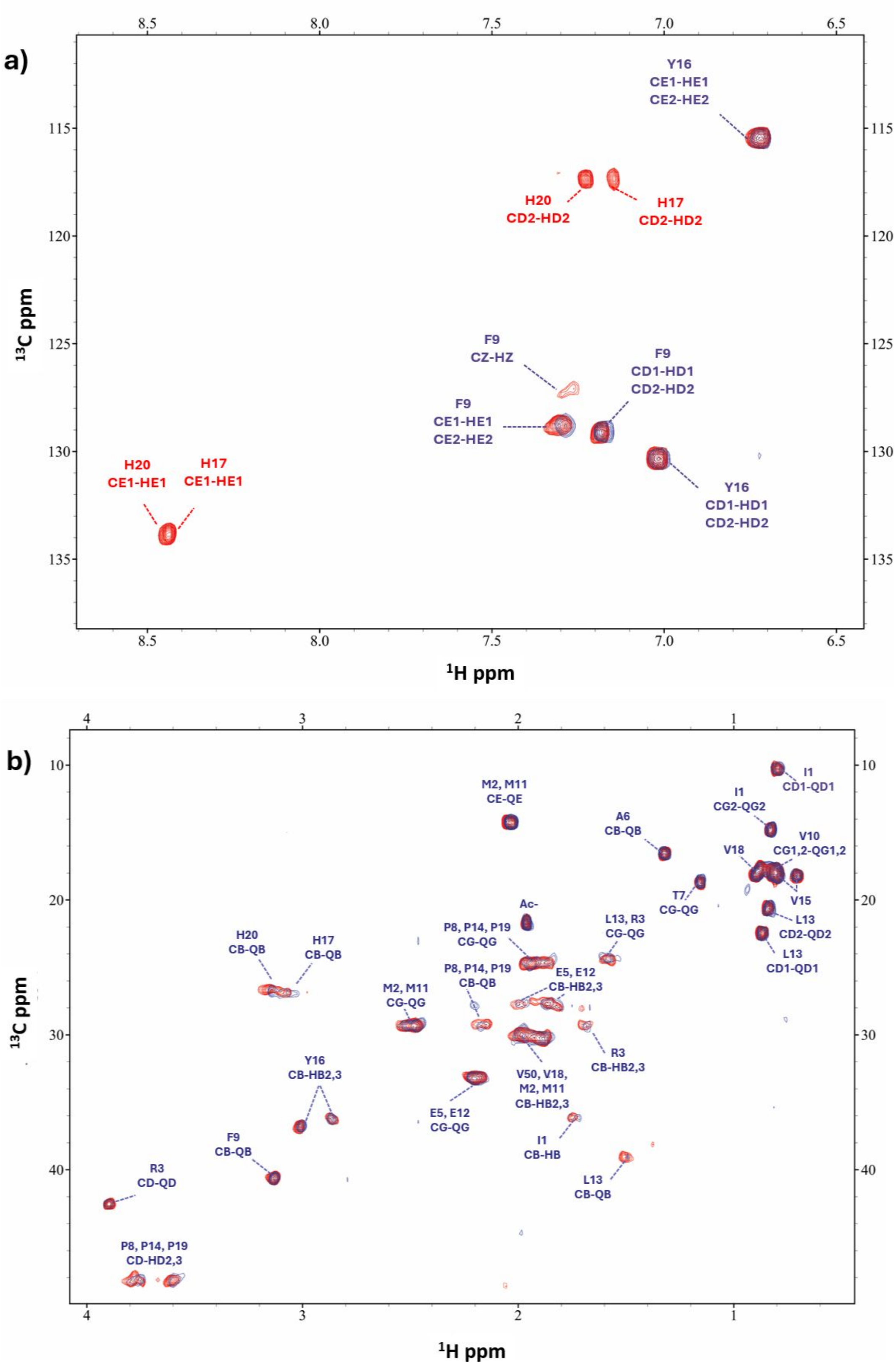

Figure S18. Comparison of a) the aromatic region and (b) aliphatic aromatic region in the ^{1}H–^{13}C HSQC spectra for the free peptide **L1** (red) and Zn(II):**L1** system (blue) at 1:1 molar ratio and pH=5.4. Perturbed signals are indicated in red.

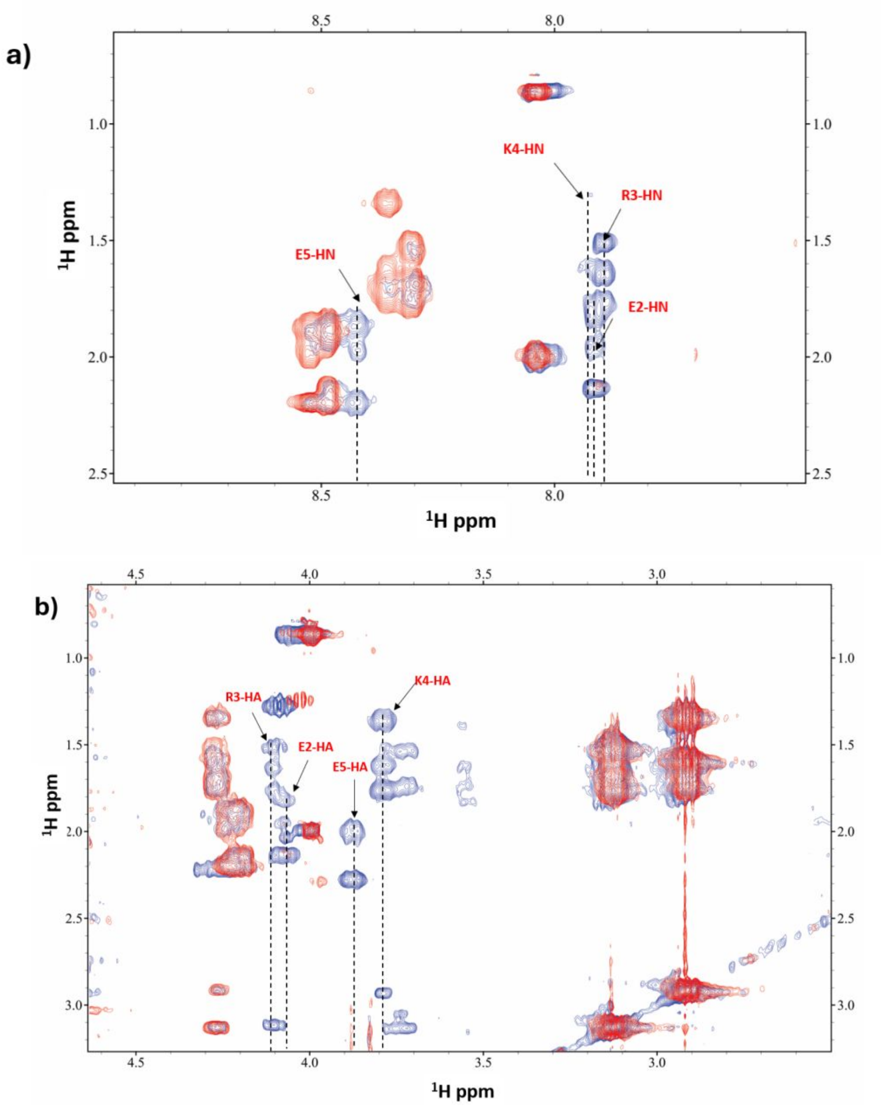

Figure S19. a) Comparison of a selected aromatic a) and b) aliphatic region in the ^{1}H−^{1}H TOCSY spectra for the free peptide **L2** (red) and Zn(II):**L2** system (blue) at 2:1 molar ratio and pH=7.0. The new set of spin system are indicated in red.

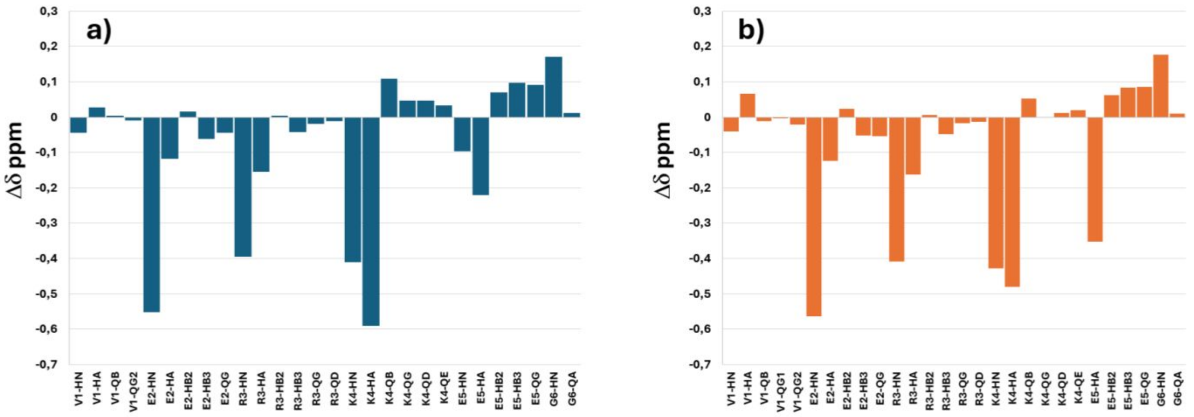

Figure S20. Chemical shift differences between Zn(II):**L2** system and free **L2** at pH=5.1 (a) and pH=7.0 (b).
